# Distribution and Drug Resistance of Bacterial Pathogens Associated with Lower Respiratory Tract Infection in Children and the Effect of COVID-19 on the Distribution of Pathogens

**DOI:** 10.1155/2022/1181283

**Published:** 2022-03-29

**Authors:** Xuan Zhu, Ting Ye, Hong Zhong, Yaxuan Luo, Jian Xu, Qin Zhang, Xiaobo Luo, Qin Wang, Liyuan Zhang, Peipei Song, Jun Zhang

**Affiliations:** ^1^Department of Clinical Laboratory, Chengdu Women's and Children's Central Hospital, School of Medicine, University of Electronic Science and Technology of China, Chengdu 611731, China; ^2^Key Laboratory of Diagnostic Medicine Designated by the Chinese Ministry of Education, Chongqing Medical University, Chongqing 400016, China; ^3^Department of Pediatric Hematology and Oncology Department, Chengdu Women's and Children's Central Hospital, School of Medicine, University of Electronic Science and Technology of China, Chengdu 611731, China

## Abstract

By studying the distribution and drug resistance of bacterial pathogens associated with lower respiratory tract infection (LRTI) in children in Chengdu and the effect of the COVID-19 on the distribution of pathogens and by analyzing the epidemic trend and drug resistance changes of the main pathogens of LRTI, this research is supposed to provide a useful basis for the prevention of LRTI in children and the rational use of drugs in clinical practice. Hospitalized children clinically diagnosed with LRTI in Chengdu Women and Children's Central Hospital from 2011 to 2020 were selected as the study subjects. The pathogens of LRTI in children and the distribution of pathogens in different ages, genders, seasons, years, and departments and before and after the pandemic situation of COVID-19 were counted. The drug resistance distribution of the top six pathogens with the highest infection rate in the past three years and the trend of drug resistance in the past decade were analyzed. A total of 26,469 pathogens were isolated. Among them, 6240 strains (23.6%) were Gram-positive bacteria, 20152 strains (76.1%) were Gram-negative bacteria, and 73 strains (0.3%) were fungi. *Klebsiella pneumoniae*, *Escherichia coli*, *Enterobacter cloacae*, *and Staphylococcus aureus* were highly isolated in the group of infants aged 0-1 (*P* < 0.01), *Moraxella catarrhalis* and *Streptococcus pneumoniae* were highly isolated in children aged 1–6 (*P* < 0.01), and *Haemophilus influenzae* was highly isolated in children over 1 (*P* < 0.01). The isolation rates of *Enterobacteriaceae*, *Acinetobacter baumannii*, *Pseudomonas aeruginosa*, *Stenotrophomonas maltophilia*, *Staphylococcus aureus,* and *Candida albicans* in the lower respiratory tract of 0-1 year-old male infants were higher than those of female infants (*p* < 0.05). *Haemophilus influenzae* was highly isolated in spring and summer, and *Moraxella catarrhalis* was highly isolated in autumn and winter, while the infection of *Streptococcus pneumoniae* was mainly concentrated in winter. This difference was statistically significant (*P* < 0.01). Affected by the COVID-19 pandemic, the isolation rates of *Haemophilus influenzae* and *Streptococcus pneumoniae* were significantly lower than those before the pandemic, and the isolation rate of *Moraxella catarrhalis* was significantly higher. The difference was statistically significant (*P* < 0.01). The proportion of isolated negative bacteria in NICU and PICU was higher than that in positive bacteria, and the infection rates of *Klebsiella pneumoniae*, *Escherichia coli*, *Enterobacter cloacae,* and *Acinetobacter baumannii* were higher than those in other departments. The differences were statistically significant (*P* < 0.01). The results of drug sensitivity test showed that the drug resistance of *Haemophilus influenzae* and *Moraxella catarrhalis* was mainly concentrated in Ampicillin, First- and Second-generation cephalosporins, and Cotrimoxazole, with stable sensitivity to Third-generation cephalosporins, while the drug resistance of *Streptococcus pneumoniae* was concentrated in Macrolides, Sulfonamides, and Tetracyclines, with stable sensitivity to Penicillin. *Staphylococcus aureus* is highly resistant to penicillins and macrolides and susceptible to vancomycin. *Enterobacteriaceae* resistance is concentrated in cephalosporins, with a low rate of carbapenem resistance. From 2018 to 2020, 1557 strains of *Staphylococcus aureus* were isolated, of which 416 strains were MRSA, accounting for 27% of the isolates; 1064 strains of *Escherichia coli* were isolated, of which 423 strains were ESBL and 23 strains were CRE, accounting for 40% and 2% of the isolates, respectively; and 1400 strains of *Klebsiella pneumoniae* were isolated, of which 385 strains were ESBL and 402 strains were CRE, accounting for 28% and 29% of the isolates, respectively. Since 2011, the resistance of *Escherichia coli* and *Klebsiella pneumoniae* to Third-generation cephalosporins has increased, peaking in 2017, and has decreased after 2018, years after which carbapenem resistance has increased significantly, corresponding to an increase in the detection rate of *Carbapenem-resistant Enterobacteriaceae* CRE. Findings from this study revealed that there are significant differences in community-associated infectious pathogens before and after the COVID-19 pandemic, and there are significant age differences, seasonal epidemic trends, and high departmental correlation of pathogens related to lower respiratory tract disease infection in children. There was a significant gender difference in the isolation rate of pathogens associated with LRTI in infants under one year. Vaccination, implementation of isolation measures and social distance, strengthening of personal protective measures, aseptic operation of invasive medical treatment, hand hygiene, and environmental disinfection are beneficial to reducing community-associated pathogen infection, opportunistic pathogen infection, and an increase in resistant bacteria. The strengthening of bacterial culture of lower respiratory tract samples by pediatricians is conducive to the diagnosis of respiratory tract infections caused by different pathogens, contributing to the selection of effective drugs for treatment according to drug susceptibility results, which is important for the rational use of antibiotics and curbing bacterial resistance.

## 1. Introduction

Lower respiratory tract infection LRTI is a leading cause of morbidity and mortality in the global population [1]. The Lancet 2016 Global Burden of Disease, Injury, and Risk Factors Report GBD assessed the deaths and causes from 26-year cases in 195 countries and concluded that LRTI is the leading cause of death in children under 5 and in the elderly over 70 and that *Streptococcus pneumoniae* infection is the most important factor of death in the all-age LRTI population [2]. Acute lower respiratory tract infection (ALRTI) is also affected by factors such as age, gender, medical level, sanitary conditions, air quality, and geographical economy, causing a serious burden on global health services [3, 4]. The pandemic of COVID-19 broken out in 2019 poses an unprecedented challenge to global public health safety and further places an additional burden on global health care. The prognosis of patients with SARS-CoV-2 is related to the severity of lower respiratory tract infections caused by SARS-CoV-2, with severe respiratory insufficiency [5]. Although the probability of COVID-19 of infection in children is similar to that in adults, and most children are asymptomatic infected [6], the pandemic brings a series of negative effects, including the potential destruction of medical resources and the supply shortage of antibiotics and food, which indirectly increases child mortality [7]. In particular, children in low- and middle-income countries, who account for a relatively high proportion of the population, coupled with the scarcity of medical resources, are at risk of ALRTI [8]. Analysis of the changes in lower respiratory tract bacteria in children caused by the pandemic is essential to adjust public health measures during the pandemic and to reduce the risk of LRTI and death in children brought about by the pandemic.

In recent years, with the increasing of antibiotic resistance, the improvement of life expectancy in low- and middle-income countries has been limited. Drug-resistant bacteria in health care and community settings pose a threat to the rate of survival from serious bacterial infections, including neonatal sepsis, hospital-associated infections, surgery, organ transplantation, and cancer treatment. The development of new antibiotics and the rational use of existing antibiotics have become another major challenge in the global health. It is important to assess the burden of treatment failure due to antibiotic resistance in disease and the impact of vaccines in limiting the need for antibiotic application [9]. Analysis of the distribution and epidemic characteristics of LRTI bacterial pathogens in children in recent years as well as antibacterial drug resistance and trends has important reference value for the correct clinical selection of antibacterial drugs to develop an empirical treatment plan, achieve a reasonable socioeconomic benefit ratio, and reduce the occurrence of drug-resistant strains.

The purpose of this study was to retrospectively investigate and statistically analyze the qualified sputum, endotracheal tube, bronchoalveolar lavage fluid, and other lower respiratory tract bacterial culture samples from hospitalized children with LRTI from January 2011 to December 2020 in Chengdu Women's and Children's Central Hospital and dynamically analyze the distribution, epidemic trend, and drug sensitivity results of isolated pathogens, providing a favorable basis for preventing lower respiratory tract bacterial infection in children and guiding the rational use of drugs in clinical practice.

## 2. Material and Methods

### 2.1. Study Design and Settings

This is a retrospective study; data were extracted from the electronic medical records of Chengdu Women's and Children's Central Hospital. The study objects are the pathogens isolated from the lower respiratory tract samples of pediatric departments from January 2011 to December 2020 and the first strain isolated from each child was selected while the repeated strains obtained from the same case and the same site were eliminated. All study cases met the diagnostic criteria for lower respiratory tract infection-related diseases including pneumonia, bronchitis, and bronchiolitis in children. The diagnostic criteria and exclusion criteria were based on our hospital's clinical protocol and combined with the American diagnostic criteria for pneumonia in children [10].

### 2.2. Diagnostic Criteria

Symptoms are fever, cough, and wheezing. Older children may have chest pain. Infants less than 2 months of age may be afebrile and present with spitting, breath holding, or choking cough. There is persistent fever with cough for more than 3 to 5 days.

Signs are increased respiration and moist rales. Criteria for increased respiratory rate (RR) are observation for 1 minute at rest, age 0–2 months: ≥60 beats/min; age 2–12 months: ≥50 beats/min; age 1–5 years: ≥40 beats/min; age >5 years: ≥20 beats/min. With the aggravation of the disease, there may be shallow breathing, inspiratory depression of the chest wall, nasal fan, three concave signs, groaning and cyanosis, which may have irritability, malaise, drowsiness, and refusal to eat.

Imaging studies are chest radiography or chest CT examination: unilateral pulmonary infiltration, multilobar pulmonary infiltration, pleural effusion, pneumothorax, atelectasis, pulmonary necrosis, and lung abscess.

### 2.3. Exclusion Criteria

Airway disorder, noninfectious lung disease, and pulmonary tuberculosis are the exclusion criteria.

### 2.4. Sample Collection

Sputum, endotracheal tube, and bronchoalveolar lavage fluid samples were collected from children with lower respiratory tract infection. Sputum collection from children: in the morning, use normal saline to clean oral cavity and collect the expectorated deep thick sputum with a sterile sealed container for examination. The sputum collection from infants is collected by nurses using sputum absorber. Endotracheal tube: the specimen was obtained directly with a bronchial brush near the lesion. Alveolar lavage fluid: lavage the small bronchi and alveoli with no less than 140 ml of sterile normal saline. 60 ml of recovered lavage fluid contains about 1 m of secretions in the bronchial endings and alveoli. Discard the part that may be contaminated in the anterior segment, collect the rest part, and immediately submit for examination. The sample collection process was performed in strict accordance with the ISO15189 implementation standard.

### 2.5. Pathogen Culture and Identification

The collected lower respiratory tract samples were inoculated in sterile Columbia blood medium, chocolate medium, and MacConkey medium and incubated at 37°C for 24 h–72 h in an incubator containing 5%–10% CO2 under aerobic environment.

### 2.6. Identification and Drug Sensitivity Test

VITEK-2compact bacterial identification/drug sensitivity automatic microorganism detection system produced by bioMerieux France was used for identification analysis. VITEK2 GP, VITEK2 GN, VITEK2 NH, and VITEK2 YST cards were used for bacterial identification. VITEK2 AST-GP67 and VITEK2 AST-GP68 were used for drug sensitivity test of positive bacteria. VITEK2 AST-GN13, VITEK2 AST-GN04, and VITEK2 AST-GN67 were used for drug sensitivity test of negative bacteria. ATB HAEMO was used for drug sensitivity test of *H. influenzae* and *M. catarrhalis*. Minimum inhibitory concentration MIC was used for results. The results were interpreted according to the standards of the American Committee for Clinical Laboratory Standards (CLSI) 2020.

The quality control strains adopt *Escherichia coli* ATCC25922, *Pseudomonas aeruginosa* ATCC27853, *Staphylococcus aureus* ATCC25923, and *Streptococcus pneumoniae* ATCC49619. Laboratory operations were performed in strict accordance with ISO15189 procedure documents.

### 2.7. Statistical Data Analysis

WHONET5.6 was used for statistical analysis of pathogen distribution and drug sensitivity results, and Excel software was used for data processing of statistical results. The isolation rates of pathogens from different seasons and departments were compared, SPSS 19.0 statistical software was applied for statistics, and Four-table chi-square test and multiple independent sample contingency table *χ*2 tests were selected, which have statistically significant differences at *P* < 0.05.

## 3. Result

### 3.1. Distribution of Pathogens in Lower Respiratory Tract Infections

**Figure 1:**
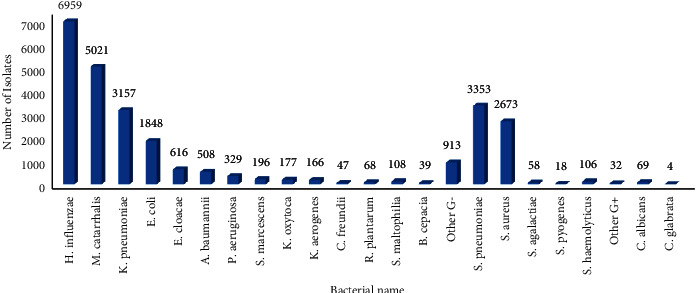
Isolated pathogens of lower respiratory tract from 2011 to 2020.

**Figure 2:**
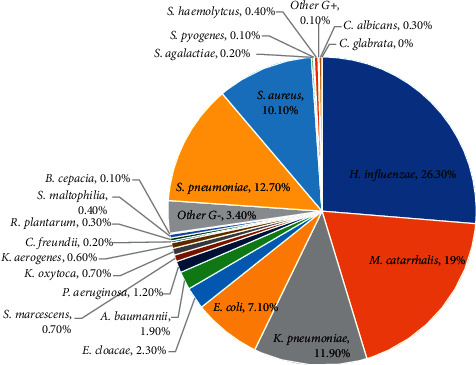
Composition ratio of pathogens isolated from lower respiratory tract from 2011 to 2020.

**Figure 3:**
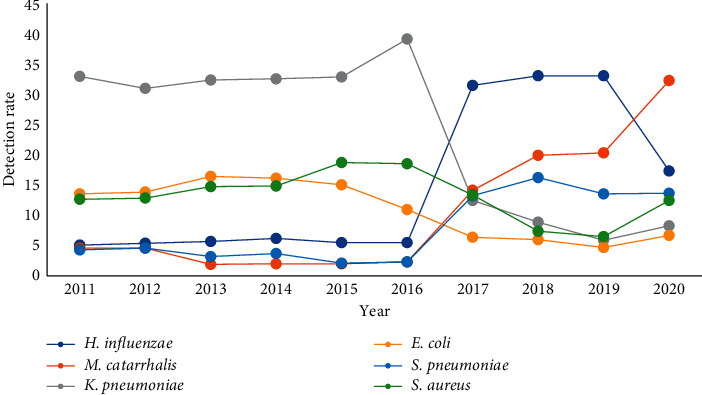
Changes in the detection rate of pathogens in lower respiratory tract from 2011 to 2020 (%).

A total of 26,469 pathogens were detected from January 2011 to December 2020. Among them, 6240 strains (23.6%) were Gram-positive bacteria, 20152 strains (76.1%) were Gram-negative bacteria, and 73 strains (0.3%) were fungi. The pathogens with the highest detection rate were *Haemophilus influenzae*, *Moraxella catarrhalis*, *Streptococcus pneumoniae*, *Staphylococcus aureus*, *Klebsiella pneumoniae*, and *Escherichia coli*. Since 2017, the proportion of opportunistic infectious pathogens mainly including *Klebsiella pneumoniae*, *Escherichia coli*, and *Staphylococcus aureus* showed a decreasing trend, while community associated infectious pathogens mainly including *Haemophilus influenzae*, *Moraxella catarrhalis*, and *Streptococcus pneumoniae* increased. The outbreak of the COVID-19 in 2019 caused some impact on the pathogen structure, Figures 1–3. (The data of Figures 1–3 originate from Supplementary Material Table 1.)

### 3.2. Pathogens of Lower Respiratory Tract Infection and Their Gender and Age Distribution

**Figure 4:**
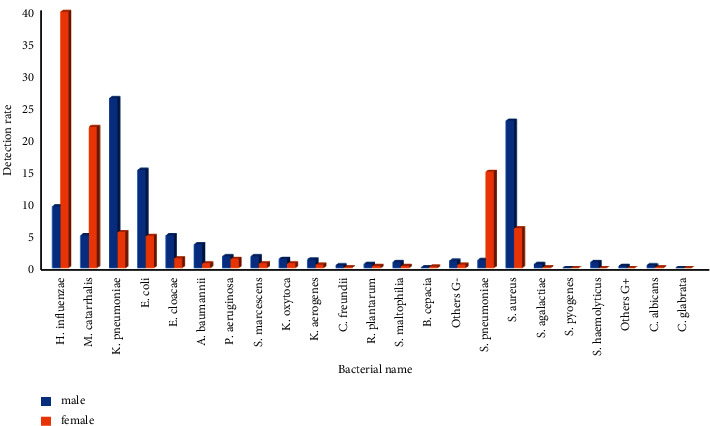
Detection rate of lower respiratory tract pathogens by gender in infant group (0-1 year old) from 2011 to 2020 (%).

**Figure 5:**
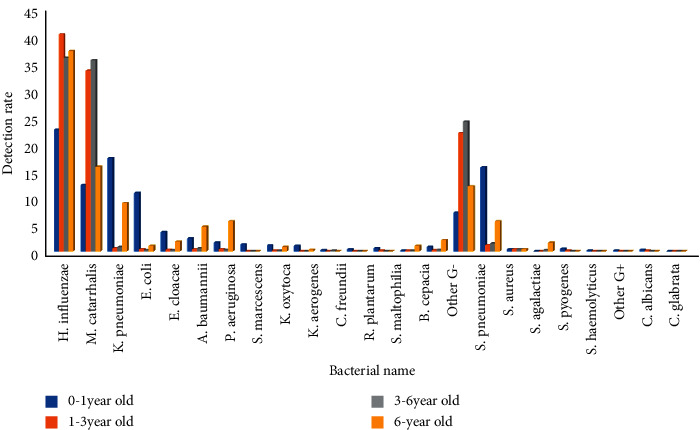
Detection rate of lower respiratory tract pathogens in different age groups from 2011 to 2020 (%).

Pathogens isolated from the lower respiratory tract had gender differences only in the 0-1-year-old infant group and no differences in the other groups. The isolation rates of *Klebsiella pneumoniae*, *Escherichia coli*, *Enterobacter cloacae*, *Serratia marcescens*, *Klebsiella oxytoca*, *Enterobacter aerogenes*, *Citrobacter freundii*, *Raoultella plantarum*, *Acinetobacter baumannii*, *Pseudomonas aeruginosa*, *Stenotrophomonas maltophilia*, *Staphylococcus aureus,* and *Candida albicans* in male infants aged 0-1 years were higher than those in female infants, the isolation rates were 26.3%, 15.2%, 5.0%, 1.8%, 1.4%, 1.3%, 0.4%, 0.6, 3.6%, 1.8%, 0.9%, 22.8%, and 0.4%, respectively, and the differences were statistically significant (*p* < 0.05). The detection rates of *Haemophilus influenzae*, *Moraxella catarrhalis,* and *Streptococcus pneumoniae* in male infants were lower than those in female infants, the isolation rates were 9.5%, 5.0%, and 1.2%, respectively, and the differences were statistically significant (*p* < 0.01). Among the age groups, *Klebsiella pneumoniae*, *Escherichia coli*, *Enterobacter cloacae,* and *Staphylococcus aureus* were highly isolated in the 0-1-year-old infant group, 17.3%, 10.8%, 3.5%, and 15.6%, respectively, and the difference was statistically significant (*P* < 0.01)(*P* < 0.01). *Moraxella catarrhalis* and *Streptococcus pneumoniae* were highly isolated in 1–3-year-old toddler and 3–6-year-old preschool children, with isolation rates of 33.5%, 35.5%, and 22.0%, 24.0%, respectively, and the difference was statistically significant (*P* < 0.01). *Haemophilus influenzae* was highly isolated in children over 1 year of age, with isolation rates of 40.3% in toddler, 35.9% in preschool age, and 37.2% in school age, respectively, and the difference was statistically significant (*P* < 0.01), Figures 4 and 5. (The data of Figures 4 and 5 originate from Supplementary Material Table 2.)

### 3.3. Pathogens and Seasonal Distribution of Lower Respiratory Tract Infection

**Figure 6:**
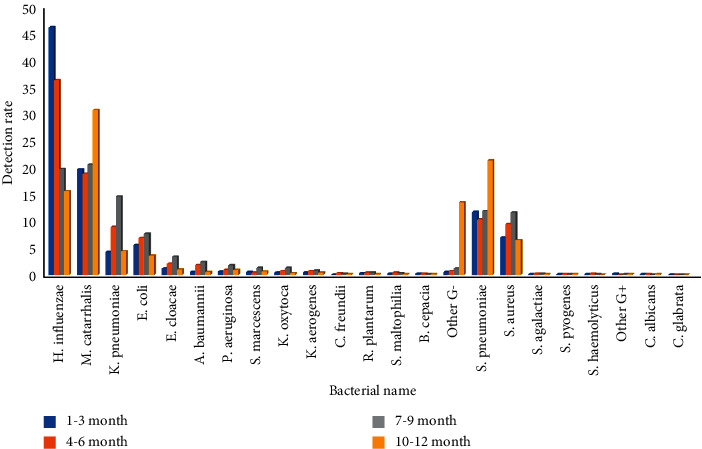
Detection rate of pathogens in lower respiratory tract in different seasons from 2018 to 2020 (%).

Among the pathogens isolated in different seasons, *Haemophilus influenzae* had a higher isolation rate in the first and second quarters, 46.4% and 36.5%, respectively, and the difference was statistically significant (*P* < 0.01); *Moraxella catarrhalis* had a higher isolation rate in the third and fourth quarters, 20.6% and 30.9%, respectively, and the difference was statistically significant (*P* < 0.01); *Streptococcus pneumoniae* had a higher isolation rate in the fourth quarter, reaching 21.4% and the difference was statistically significant (*P* < 0.01), Figure 6. (The data of Figure 6 originate from Supplementary Material Table 3.)

### 3.4. Pathogens of Lower Respiratory Tract Infection and Pandemic of COVID-19 Situation

**Figure 7:**
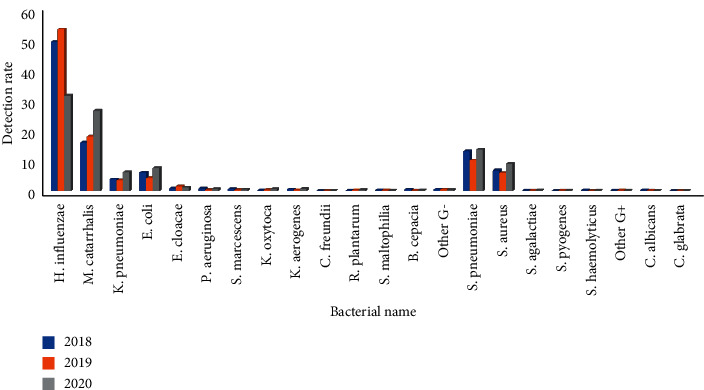
Detection rate of pathogens in lower respiratory tract in the first quarter before and after the pandemic situation of COVID-19 (%).

**Figure 8:**
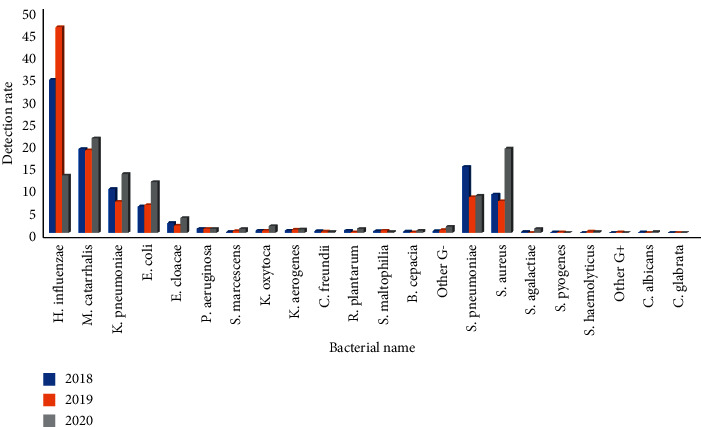
Detection rate of pathogens in lower respiratory tract in the second quarter before and after the pandemic situation of COVID-19 (%).

**Figure 9:**
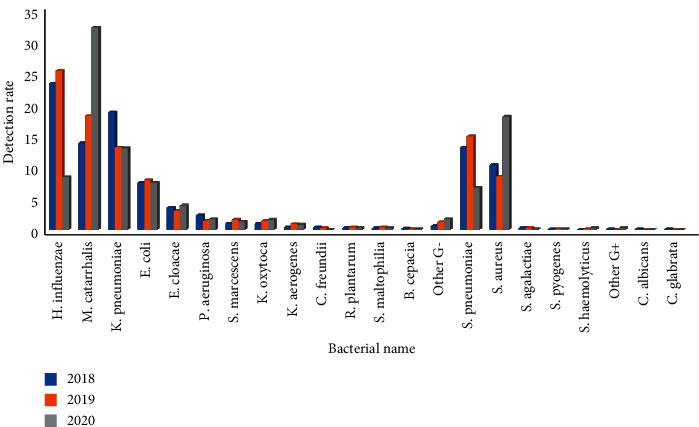
Detection rate of pathogens in lower respiratory tract in the third quarter before and after the pandemic situation of COVID-19 (%).

**Figure 10:**
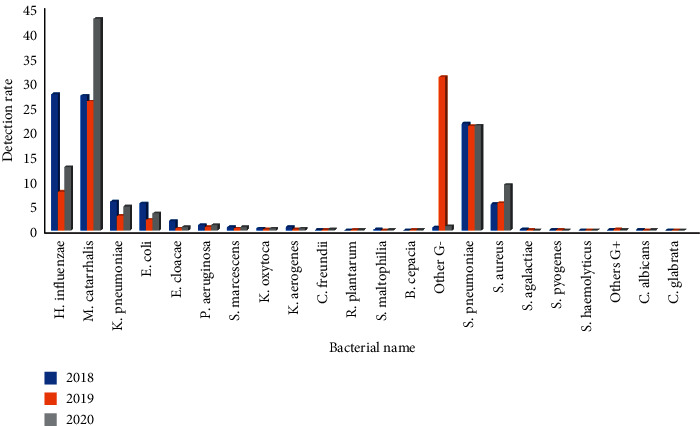
Detection rate of pathogens in lower respiratory tract in the fourth quarter before and after the pandemic situation of COVID-19 (%).

From 2018 to 2020, the first-quarter infection rate of *Haemophilus influenzae* was 49.3%, 53.3%, and 31.5%, respectively, and the difference had statistical significance (*P* < 0.01)(*P* < 0.01). The second quarter infection rate was 34.1%, 45.9%, and 12.8%, respectively, and the difference had statistical significance (*P* < 0.01). The third-quarter infection rate was 23.2%, 25.2%, and 8.3%, respectively, and the difference had statistical significance (*P* < 0.01). The fourth quarter infection rate was 27.7%, 7.8%, and 12.8%, respectively, and the difference had statistical significance (*P* < 0.01). The infection rate of *Moraxella catarrhalis* in the first quarter was 15.9%, 17.9%, and 26.5%, respectively, and the difference had statistical significance (*P* < 0.01); the infection rate in the second quarter was 18.7%, 18.4%, and 21.1%, respectively, and the difference had no statistical significance (*P* > 0.05); the infection rate in the third quarter was 13.7%, 18.0%, and 32.0%, respectively, and the difference had statistical significance (*P* < 0.01); the infection rate in the fourth quarter was 27.4%, 26.2% and 43.0%, respectively, and the difference had statistical significance (*P* < 0.01). The first-quarter infection rate of *Streptococcus pneumoniae* was 13.0%, 10.0%, and 13.6%, respectively, and the difference had statistical significance (*P* < 0.01). The second quarter infection rate was 14.7%, 7.9%, and 8.2%, respectively, and the difference had statistical significance (*P* < 0.01). The third-quarter infection rate was 13.0%, 14.8%, and 6.6%, respectively, and the difference had statistical significance (*P* < 0.01). The fourth quarter infection rate was 21.7%, 21.2%, and 21.3%, respectively, and the difference had no statistical significance (*P* < 0.01), Figures 7–10. (The data of Figures 7–10 originate from Supplementary Material Table 4.)

### 3.5. Pathogens and Department Distribution of Lower Respiratory Tract Infection

**Figure 11:**
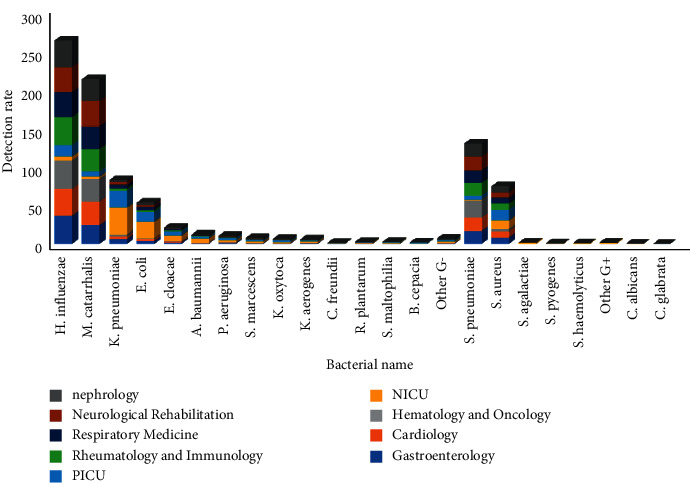
Distribution and constituent ratio of pathogens in lower respiratory tract in different departments isolated from 2018 to 2020 (%).

The isolated strains in each department showed that the infection rate of Gram-negative bacteria in PICU and NICU was higher than that in other departments, 85.1% and 79.9%, respectively, and the difference had statistical significance (*P* < 0.01). The infection rate of Gram-positive bacteria was lower than that in other departments, 14.8% and 19.8%, respectively, and the difference had statistical significance (*P* < 0.01). Opportunistic infection-related pathogens: the infection rate of *Klebsiella pneumoniae*, *Escherichia coli,* and *Staphylococcus aureus* in NICU was 34.4%, 20.7%, and 10.9%, respectively, which was significantly different from that in other departments (*P* < 0.01, *P* < 0.01, *P* < 0.01). The infection rate of *Klebsiella pneumoniae*, *Escherichia coli,* and *Staphylococcus aureus* in PICU was 22.1%, 12.6%, and 14.3%, respectively, which was significantly different from that in other departments (*P* < 0.01, *P* < 0.01, *P* < 0.01). Pathogens of invasive medical and catheter-related infections: there was significant difference in the infection rate of *Acinetobacter baumannii* in NICU (5.6%) (*P* < 0.01), and there was significant difference in the infection rate of *Acinetobacter baumannii* in PICU (2.3%) (*P* < 0.01). Community-associated infection pathogens: the infection rate of *Haemophilus influenzae*, *Moraxella catarrhalis,* and *Streptococcus pneumoniae* in NICU was 5.1%, 2.6%, and 0.6%, respectively, which were significantly different from those in other departments (*P* < 0.01, *P* < 0.01, *P* < 0.01). The infection rate of *Haemophilus influenzae*, *Moraxella catarrhalis,* and *Streptococcus pneumoniae* in PICU was 15.2%, 7.3%, and 5.5%, respectively, which were significantly different from those in other departments (*P* < 0.01, *P* < 0.01, *P* < 0.01), Figure 11. (The data of Figure 11 originate from Supplementary Material Table 5.)

### 3.6. Analysis of Drug Resistance of *Streptococcus pneumoniae*

**Figure 12:**
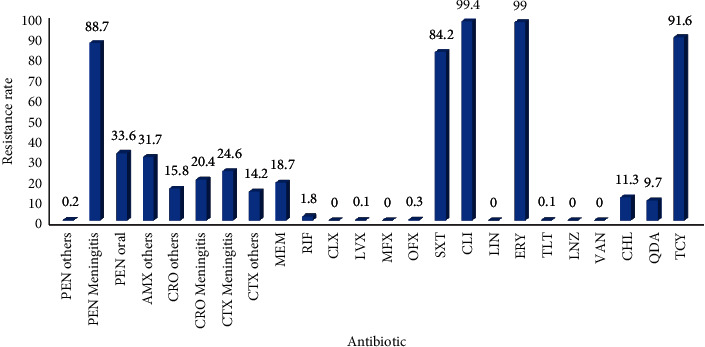
Distribution of drug resistance of *S. pneumoniae* from 2018 to 2020 (%).

**Figure 13:**
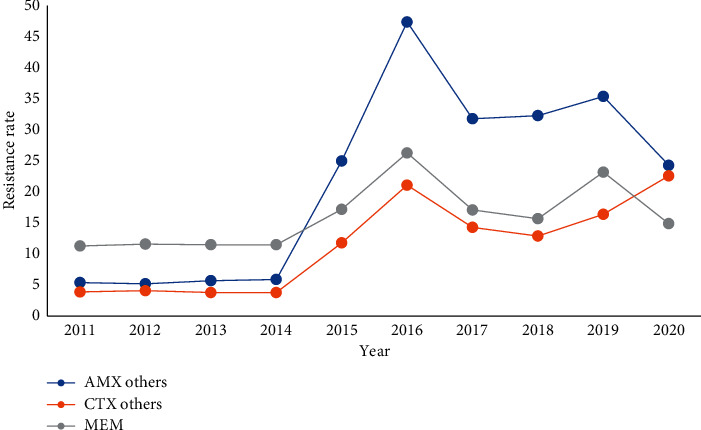
Changes in drug resistance of *S. pneumoniae* from 2011 to 2020 (%).

The sensitivity of *Streptococcus pneumoniae* strains isolated from the lower respiratory tract to Penicillin was stable and the drug resistance was mainly concentrated in Cotrimoxazole, Clindamycin, Erythromycin, and Tetracycline, with resistance rates of 84.2%, 99.4%, 99%, and 91.6%, respectively. Since 2015, the resistance rate of *Streptococcus pneumoniae* to Amoxicillin, Third-generation cephalosporins, and Meropenem has been increasing year by year, Figures 12 and 13. (The data of Figures 12 and 13 originate from Tables 6 and 7 in Supplementary Material, respectively.)

### 3.7. Analysis of Drug Resistance of *Staphylococcus aureus*

**Figure 14:**
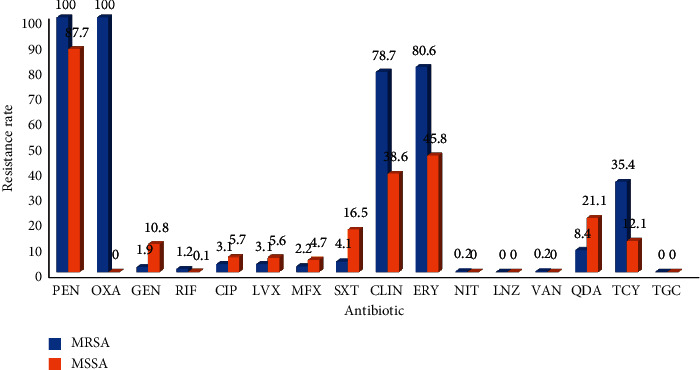
Distribution of drug resistance of *S. aureus* from 2018 to 2020 (%).

**Figure 15:**
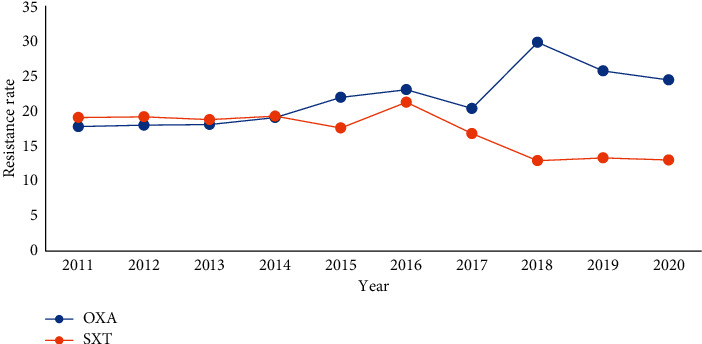
Changes in drug resistance of *S. aureus* from 2011 to 2020 (%).

The resistance rates of *Staphylococcus aureus* to Penicillin, Clindamycin, and Erythromycin were high: the resistance rates of MRSA were 100%, 78.7%, and 80.6%, respectively, and the resistance rates of MSSA were 87.7%, 38.6%, and 45.8%, respectively. Since 2011, Methicillin-resistance of *Staphylococcus aureus* has increased, the detection rate of relatively *Methicillin-resistant staphylococci* (MRSA) has been increasing year by year, and after 2015, resistance of *Staphylococcus aureus* to Cotrimoxazole has decreased, Figures 14 and 15. (The data of Figures 14 and 15 originate from Tables 8 and 9 in Supplementary Material, respectively.)

### 3.8. Analysis of Drug Resistance of *Haemophilus influenzae* and *Moraxella catarrhalis*

**Figure 16:**
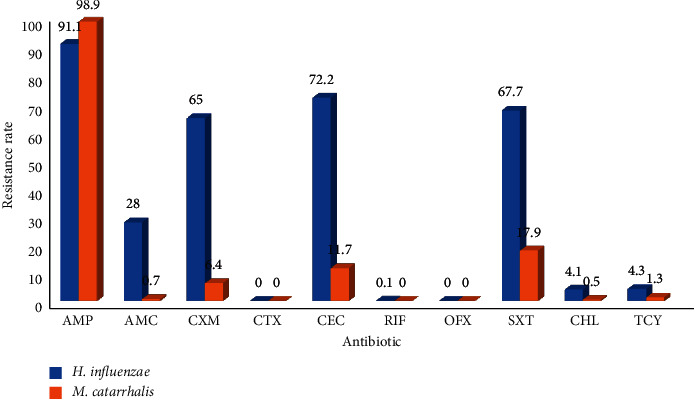
Distribution of drug resistance of *H. influenzae* and *M. catarrhalis* from 2018 to 2020 (%).

**Figure 17:**
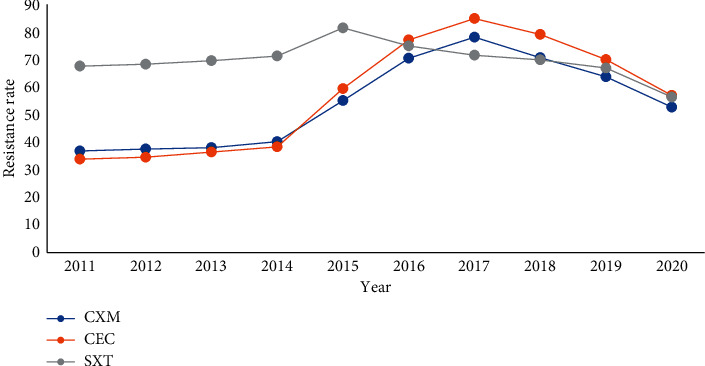
Changes in drug resistance of *H. influenzae* from 2011 to 2020 (%).

**Figure 18:**
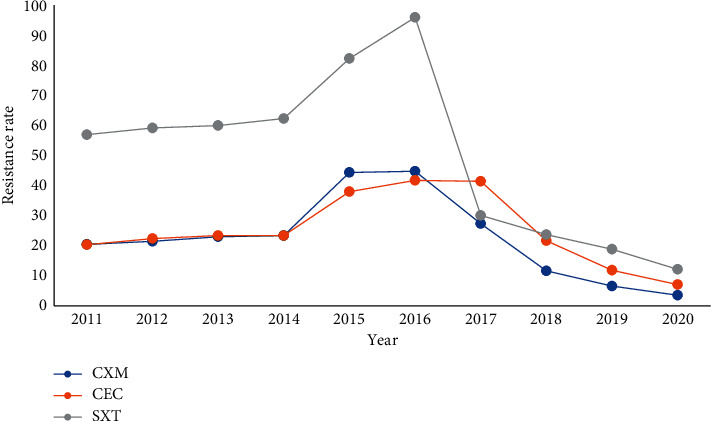
Change in drug resistance of *M. catarrhalis* from 2011 to 2020 (%).

Drug resistance of *Haemophilus influenzae* was concentrated in Ampicillin, First- and Second-generation cephalosporins, and Cotrimoxazole, while *Moraxella catarrhalis* was mainly resistant to Ampicillin, and the sensitivity of the Second- to Third-generation cephalosporins was stable. From 2011 to 2017, the resistance of *Haemophilus influenzae* and *Moraxella catarrhalis* to Second-generation cephalosporins and Cotrimoxazole increased year by year and decreased from 2018 to 2020, Figures 16–18. (The data of Figures 16–18 originate from Tables 10–12 in Supplementary Material, respectively.)

### 3.9. Analysis of Drug Resistance of *Escherichia coli* and *Klebsiella pneumoniae*

**Figure 19:**
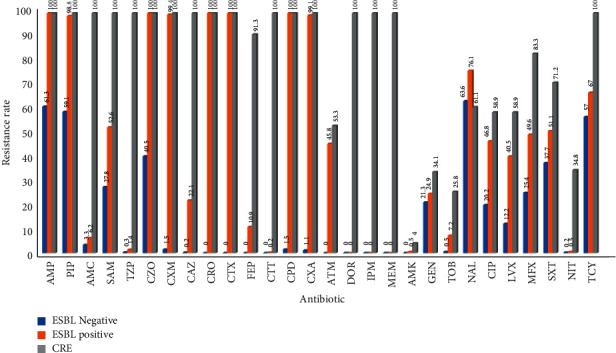
Distribution of drug resistance of *E. coli* from 2018 to 2020 (%).

**Figure 20:**
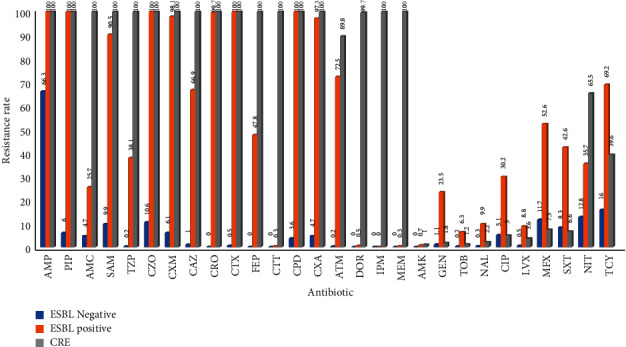
Distribution of drug resistance of *K. pneumoniae* from 2018 to 2020 (%).

**Figure 21:**
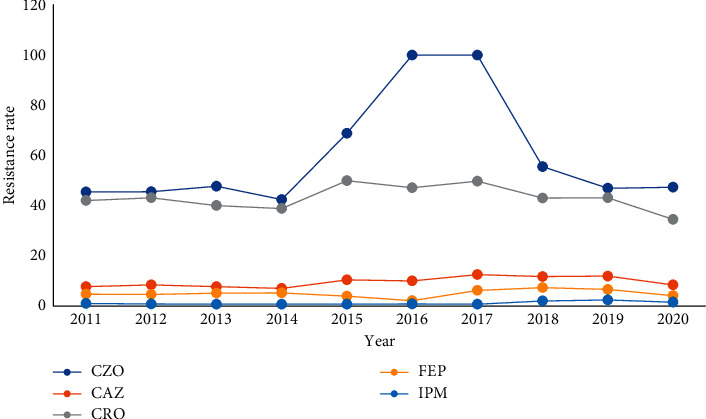
Changes in drug resistance of *E.coli* from 2011 to 2020 (%).

**Figure 22:**
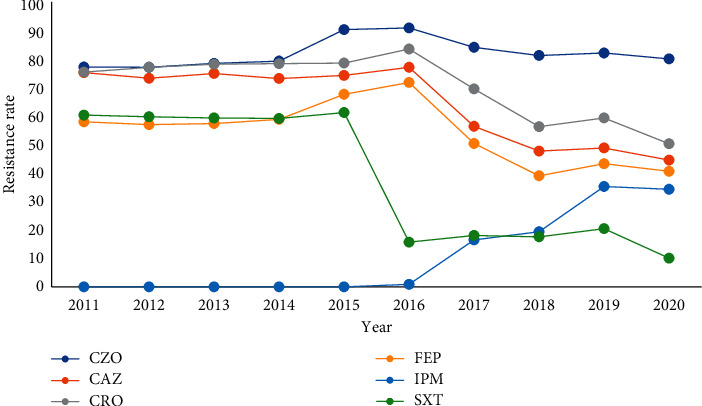
Changes in drug resistance of *K. pneumoniae* from 2011 to 2020 (%).

ESBL-negative *Escherichia coli* and *Klebsiella pneumoniae* showed high resistance to Penicillin antibiotics, while being sensitive to Cephalosporins; ESBL-positive *Escherichia coli* and *Klebsiella pneumoniae* showed severe resistance to Penicillin and First-, Second-, and Third-generation cephalosporins, while being sensitive to Fourth-generation cephalosporins and Carbapenems and some strains could be inhibited by enzyme inhibitors; CRE showed high resistance to *β*-lactam antibiotics and Carbapenems. According to the drug resistance trend diagram, the resistance of *Escherichia coli* and *Klebsiella pneumoniae* to Penicillins has remained at a high level, but decreased after 2018. The resistance to Cephalosporins has been increasing year by year, peaking at 2016 and 2017, and has gradually decreased after 2018. After 2016, the resistance rate of *Klebsiella pneumoniae* to Cotrimoxazole showed a decreasing trend year by year. The resistance of *l Escherichia coli* and *Klebsiella pneumoniae* to Carbapenems was rare before 2015 and increased after 2016, and the detection rate of CRE has been increasing year by year, Figures 19–22. (The data of Figures 19–22 originate from Tables 13–15 in Supplementary Material, respectively.)

## 4. Discussion

Lower respiratory tract infections remain to be a public threat to children in developing countries and a major infectious cause of death. Understanding the geographic patterns and potential risks of the disease can help to improve community-based treatments and interventions, thereby reducing child mortality [11]. The etiology of LRTI includes viral pathogens and bacterial pathogens [12], while severe lower respiratory tract infection ALRTI is considered to be one of the precursors of chronic bacterial bronchitis, chronic wet cough, and chronic suppurative lung disease [13]. With the outbreak of global COVID-19, the prognosis of COVID-19 patients depends on the severity of lower respiratory tract infections caused by severe acute respiratory syndrome coronavirus 2 SARS-CoV-2 [5]. Timely understanding of the distribution and epidemic trend of bacterial pathogens in the lower respiratory tract is conducive to improving the implementation of prevention, treatment, and management of lower respiratory tract infections in the community.

Improving the quality of antibiotic use is a major goal of the WHO Global Action to Fight Microbial Resistance, and studies have found dramatic differences in the proportion of AWaRe antibiotics used among hospitalized neonates and children occurred worldwide [14]. Reports from the United Kingdom have shown that antibiotics are used much longer than recommended by guidelines during primary care, resulting in antibiotic overexposure [15], while another report from Europe has shown that one-third of prescriptions for antibiotics in febrile children are unreasonable or uncertain and that antibiotic management should be implemented to limit antibiotic use before distinguishing bacterial from viral etiologies [16]. Therefore, it is particularly important to analyze the distribution of drug resistance of lower respiratory tract pathogens, so as to guide clinicians to use empirical drugs before determining the cause of lower respiratory tract infection and reduce the irrational use of antibiotics.

The results of this retrospective analysis showed that the six pathogens with the highest detection rate of lower respiratory tract in children from 2011 to 2020 were *Haemophilus influenzae*, *Moraxella catarrhalis*, *Streptococcus pneumoniae*, *Staphylococcus aureus*, *Klebsiella pneumoniae,* and *Escherichia coli*. *Haemophilus influenzae*, *Moraxella catarrhalis,* and *Streptococcus pneumoniae* are also the top three pathogens of throat colonization rate in children. At the same time, there is a synergistic relationship between these three bacteria, and the carrier density is positively correlated with each other [17]. Bacteria that colonize the upper respiratory tract of young children play a key role in the pathogenesis of LRTI [18], and studies from Australia have shown that the incidence of ALRTI is positively correlated with the detection rate of colonizing bacteria at the rhinitis site in children [19].

From the detection rate of pathogens in different age groups, it can be easily found that the probability of community-acquired pneumonia CAP is higher in the age group of 1–6, which is closely related to the high colonization rate of *Haemophilus influenzae*, *Moraxella catarrhalis,* and *Streptococcus pneumoniae* in young children [20, 21]. There was a gender gap in the isolation rate of lower respiratory tract pathogens only in the infant group, and male infants were more likely to cause ALRI than female infants due to infection with opportunistic pathogens and catheter-related pathogens. A study from India demonstrated that in developing countries, boys under 5 years of age had a higher incidence of ALRI than girls, and boys hospitalized for ALRI were 2.4 times more likely than girls [22].

Influenced by seasonal factors, *Haemophilus influenzae* was detected more frequently in spring, and *Streptococcus pneumoniae* and *Moraxella catarrhalis* were detected more frequently in winter. In low- and middle-income countries, pneumonia is the main infectious pathogen disease burden in the community, and after the introduction of 10-valent pneumococcal nontypeable *Haemophilus influenzae* protein D conjugate vaccine PCV10 in Kilifi County Hospital, Kenya, the number of pneumonia hospitalizations decreased dramatically, and the burden of pneumonia hospitalizations in children was greatly reduced [23]. China, as a country with high antibiotic use, has low PCV coverage, while studies have shown that increasing PCV coverage can reduce colonization of upper respiratory tract pathogens [24]and antibiotic resistance rates [25]. In addition, the relief of burden brought about by vaccines is not limited to ALRTI but also includes COVID-19, and influenza vaccine and pneumonia vaccine applications can synergistically prevent COVID-19 [26]. Therefore, vaccination against *Streptococcus pneumoniae* and *Haemophilus influenzae* in autumn and winter is an effective way to reduce the incidence and mortality of lower respiratory tract infections in children [27].

Through the comparison of the pathogen detection rates in the four quarters three years before and after COVID-19, it was found that the detection rates of *Haemophilus influenzae* and *Streptococcus pneumoniae* were significantly lower during the epidemic. In the third and fourth quarters of 2020, the epidemic situation was comprehensively controlled in China, and the epidemic-related measures were gradually relaxed, along with which the detection rate of *Streptococcus pneumoniae* has returned to the normal level in the fourth quarter, while the increased detection rate of *Moraxella catarrhalis* during the pandemic may be related to its bacterial structure changes during respiratory colonization and the epidemic. In conclusion, social distance and personal protection-related measures especially the use of masks can effectively reduce infectious pathogen infections in the community, to a certain extent, reducing lower respiratory tract infections caused by air pollution [28] and PM2.5 [29]. It could also serve as a community management experience for improving children's health in developing countries.

The difference in pathogen distribution among pediatric departments is mainly reflected in the intensive care unit. The detection rates of opportunistic infection-related pathogens such as *Escherichia coli*, *Klebsiella pneumoniae*, *Staphylococcus aureus* and invasive medical-related pathogens *Acinetobacter baumannii* in NICU and PICU were higher than those in other departments and the community associated infectious pathogens were lower than that in other departments. Compared with the results of big data statistics on the pathogen detection rate in the all-age intensive care unit of the Chinese Antibiotic Resistance Surveillance Network CARSS, there were differences in *Klebsiella pneumoniae*, *Pseudomonas aeruginosa*, *Acinetobacter baumannii,* and *Streptococcus pneumoniae* [30]. In addition, the detection rates of resistant bacteria ESBL-EC, ESBL-KP, and CRE were also higher than those in intensive care units of the whole age group, which were more in line with the characteristics of infection in the pediatric population [31]; therefore, management measures for pediatric-related infectious pathogens should be developed in terms of nosocomial sensing control strategies. The constituent ratio of lower respiratory tract pathogens showed that opportunistic associated infection pathogens showed a decreasing trend after 2017, and community associated infection pathogens showed an increasing trend which was inseparable from the strengthening of nosocomial infection management measures in recent years, including the reporting of drug-resistant bacteria, isolation of the patient, and environment disinfection.

Susceptibility results showed that *Haemophilus influenzae* and *Moraxella catarrhalis* resistance of infectious pathogens in the community were concentrated in ampicillin, First- and Second-generation Cephalosporins, and Cotrimoxazole which were sensitive to Third-generation cephalosporins, while *Haemophilus influenzae* bacterial strains from Iran showed high resistance rates to a variety of antibiotics [32] which may be related to regional differences in the use of antibiotics and different degrees of antibiotics exposure. *Streptococcus pneumoniae* remains persistently sensitive to Penicillin from 2011 to 2020, using the CLSI intravenous breakpoint; the sensitivity was 98%, which was higher than that in Korea and consistent with other Asian countries [33]. Then, Penicillin can be used as the first choice of drugs for the treatment of LRTI caused by *Streptococcus pneumoniae* in this region. The application of *Streptococcus pneumoniae* vaccines can reduce antibiotic use thereby reducing disease burden caused by *Streptococcus pneumoniae* resistant strains [34].

In the past three years, 73% of *Staphylococcus aureus* detected in the lower respiratory tract of children were MRSA and the detection rate of MRSA showed an increasing trend year by year with the increase of oxacillin resistance. Reports from Japan suggest that increased MRSA detection rates are associated with the rapid spread of *Staphylococcus aureus* carrying the causative gene in the environment [35], while reports from Spain showed that the use of antibiotics in animal husbandry promoted the spread of *Staphylococcus* antibiotic resistance genes in the environment and wildlife [36]. So far, Vancomycin is still the main drug for the treatment of MRSA.

Both *Escherichia coli* and *Klebsiella pneumoniae* showed a high resistance rate to Cephalosporins and before 2015, there were few Carbapenem-resistant strains of *Enterobacteriaceae* CRE, but after 2016, the CRE detection rate increased year by year. Detection of CRE strains was associated with increased antibiotic exposure and horizontal transmission of plasmids. Epidemiological surveys from China have shown that the overuse of antibiotics promotes the reproduction and transmission of antibiotic genes ARGs in the environment [37], while the prevalence of mcr-1 positive *Enterobacteriaceae* MCRPEC infections in Chinese populations is associated with the high frequency transfer of mcr-1 between bacteria [38]. In recent years, the Chinese government has been aware of the potential crisis caused by drug-resistant strains and has carried out a series of actions to curb bacterial resistance, including implementing a strict prescription drug system, preaching the harm caused by the abuse of antibiotics to the public, and limiting the massive use of antibiotics in animal husbandry and fisheries. Great achievement has been gained [39]. From the drug resistance trend diagram, *Haemophilus influenzae* and *Moraxella catarrhalis* consistently maintained high resistance to Second-generation cephalosporins and compound Sulfamethoxazole from 2011 to 2017 and decreased after 2018. The resistance of *Escherichia coli* and *Klebsiella pneumoniae* to Third-generation cephalosporins also showed a decreasing trend after 2018.

At present, it is not perfect that traditional bacterial culture provides referable diagnostic and therapeutic value for clinical practice. On the one hand, culture is time consuming and poorly sensitive; on the other hand, the rate of new antibiotic research and development is not as fast as the rate of bacterial resistance, so we urgently need to optimize current diagnosis and treatment and reduce the evolution and spread of drug resistance, and the advent of metagenomic technology has largely compensated for this flaw [40]. Metagenomic sequencing can identify LRTI pathogens faster than culture, accurately detect drug resistance genes, reduce antibiotic exposure, and, at the same time, provide the possibility to carry out precise clinical treatment and develop personalized treatment plans [41].

## 5. Conclusions

There are significant age characteristics, seasonal epidemic trends, and high departmental correlation of infection-related pathogens in children with lower respiratory tract diseases. The incidence of ALRTI is higher in male infants under one year than in female infants. Vaccination, strengthening of personal protective measures, aseptic operation of invasive medical treatment, hand hygiene, and environmental disinfection are conducive to synergistically coping with the epidemic situation of COVID-19 and reducing seasonal epidemic-related pathogen infection, opportunistic associated pathogen infection and increase of new resistant bacteria. According to the distribution and drug resistance characteristics of pathogens in this region, clinical staff use drugs rationally and improve the curative effect in combination with the results of drug sensitivity test, by adopting metagenomic detection technology when conditions permit, which can quickly and accurately obtain the information of infectious bacteria.

## Data Availability

The data used to support the findings of this study are included within the supplementary information file.

## Supplementary Materials

Supplementary Materials The corresponding data of each figure are placed in the Supplementary Material and presented in the form of tables with the same number.
