# Supplementary material for: Distribution and Drug Resistance of Bacterial Pathogens Associated with Lower Respiratory Tract Infection in Children and the Effect of COVID-19 on the Distribution of Pathogens
Source: Can J Infect Dis Med Microbiol. 2022 Mar 29;2022:1181283. doi: 10.1155/2022/1181283 (PMC8965734; doi:10.1155/2022/1181283)
Supplement: Supplementary Materials — The corresponding data of each figure are placed in the Supplementary Material and presented in the form of tables with the same number. [file 1181283.f1.docx]

**3.1 Distribution of pathogens in lower respiratory tract infections. Table1.**

Table 1 Distribution and constituent ratio of pathogens in lower respiratory tract in different years isolated from 2011 to 2020 (%)

|  | 2011 | 2012 | 2013 | 2014 | 2015 | 2016 | 2017 | 2018 | 2019 | 2020 | 2011-2020 |
| --- | --- | --- | --- | --- | --- | --- | --- | --- | --- | --- | --- |
| ***G^-^*** | 291(78.9) | 303(77.9) | 536(78.4) | 540(77.4) | 602(73.5) | 617(76.1) | 2743(72.3) | 4365(75.8) | 6775(79.4) | 3380(73.2) | 20152(76.1) |
| *H. influenzae* | 19(5.1) | 21(5.4) | 39(5.7) | 43(6.2) | 45(5.5) | 45(5.5) | 1200(31.6) | 1912(33.2) | 2832(33.2) | 803(17.4) | 6959(26.3) |
| *M. catarrhalis* | 17(4.6) | 18(4.6) | 13(1.9) | 14(2.0) | 16(2.0) | 19(2.3) | 538(14.2) | 1149(20.0) | 1743(20.4) | 1494(32.4) | 5021(19.0) |
| *K. pneumoniae* | 122(33.1) | 121(31.1) | 222(32.5) | 228(32.7) | 270(33.0) | 319(39.3) | 475(12.5) | 510(8.9) | 506(5.9) | 384(8.3) | 3157(11.9) |
| *E. coli* | 50(13.6) | 54(13.9) | 113(16.5) | 113(16.2) | 124(15.1) | 89(11.0) | 241(6.4) | 348(6.0) | 405(4.7) | 311(6.7) | 1848(7.0) |
| *E. cloacae* | 31(8.4) | 32(8.2) | 42(6.1) | 34(4.9) | 35(4.3) | 38(4.7) | 69(1.8) | 119(2.1) | 128(1.5) | 88(1.9) | 616(2.3) |
| *A. baumannii* | 26(7.0) | 29(7.5) | 52(7.6) | 51(7.3) | 42(5.1) | 35(4.3) | 53(1.4) | 80(1.4) | 78(0.9) | 62(1.3) | 508(1.9) |
| *P. aeruginosa* | 9(2.4) | 9(2.3) | 18(2.6) | 20(2.9) | 18(2.2) | 25(3.1) | 42(1.1) | 71(1.2) | 68(0.8) | 49(1.1) | 329(1.2) |
| *S. marcescens* | 4(1.1) | 4(1.0) | 4(0.6) | 4(0.6) | 13(1.6) | 11(1.4) | 30(0.8) | 36(0.6) | 53(0.6) | 37(0.8) | 196(0.7) |
| *K. oxytoca* | 3(0.8) | 4(1.0) | 5(0.7) | 5(0.7) | 5(0.6) | 8(1.0) | 32(0.8) | 27(0.5) | 47(0.6) | 41(0.9) | 177(0.7) |
| *K. aerogenes* | 6(1.6) | 6(1.5) | 8(1.2) | 9(1.3) | 7(0.9) | 6(0.7) | 20(0.5) | 31(0.5) | 43(0.5) | 30(0.6) | 166(0.6) |
| *C. freundii* | 1(0.3) | 1(0.3) | 5(0.7) | 3(0.4) | 0(0.0) | 3(0.4) | 5(0.1) | 12(0.2) | 13(0.2) | 4(0.1) | 47(0.2) |
| *R．plantarum* | 0(0.0) | 0(0.0) | 4(0.6) | 3(0.4) | 2(0.2) | 4(0.5) | 7(0.2) | 12(0.2) | 19(0.2) | 17(0.4) | 68(0.3) |
| *S. maltophilia* | 3(0.8) | 4(1.0) | 7(1.0) | 10(1.4) | 16(2.0) | 13(1.6) | 13(0.3) | 14(0.2) | 22(0.3) | 6(0.1) | 108(0.4) |
| *B. cepacia* | 0(0.0) | 0(0.0) | 0(0.0) | 0(0.0) | 0(0.0) | 1(0.1) | 9(0.2) | 12(0.2) | 8(0.1) | 9(0.2) | 39(0.1) |
| *Others* | 0(0.0) | 0(0.0) | 4(0.6) | 3(0.4) | 9(1.1) | 1(0.1) | 9(0.2) | 32(0.6) | 810(9.5) | 45(1.0) | 913(3.4) |
| ***G^+^*** | 73(19.8) | 80(20.6) | 139(20.3) | 148(21.2) | 211(25.8) | 189(23.3) | 1031(27.2) | 1383(24.0) | 1755(20.6) | 1231(26.7) | 6240(23.6) |
| *S. pneumoniae* | 16(4.3) | 18(4.6) | 22(3.2) | 26(3.7) | 17(2.1) | 19(2.3) | 503(13.3) | 938(16.3) | 1161(13.6) | 633(13.7) | 3353(12.7) |
| *S. aureus* | 47(12.7) | 50(12.9) | 101(14.8) | 104(14.9) | 154(18.8) | 151(18.6) | 509(13.4) | 424(7.4) | 558(6.5) | 575(12.5) | 2673(10.1) |
| *S.agalactiae* | 0(0.0) | 0(0.0) | 1(0.1) | 1(0.1) | 7(0.9) | 6(0.7) | 13(0.3) | 11(0.2) | 9(0.1) | 10(0.2) | 58(0.2) |
| *S. pyogenes* | 0(0.0) | 0(0.0) | 0(0.0) | 0(0.0) | 0(0.0) | 0(0.0) | 5(0.1) | 3(0.1) | 8(0.1) | 2(0.0) | 18(0.1) |
| *S. haemolyticus* | 6(1.6) | 8(2.1) | 15(2.2) | 17(2.4) | 31(3.8) | 13(1.6) | 1(0.0) | 3(0.1) | 7(0.1) | 5(0.1) | 106(0.4) |
| *Others* | 4(1.1) | 4(1.0) | 0(0.0) | 0(0.0) | 2(0.2) | 0(0.0) | 0(0.0) | 4(0.1) | 12(0.1) | 6(0.1) | 32(0.1) |
| ***Fungi*** | 5(1.4) | 6(1.5) | 9(1.3) | 10(1.4) | 6(0.7) | 5(0.6) | 18(0.5) | 9(0.2) | 3(0.0) | 2(0.0) | 73(0.3) |
| *C. albicans* | 5(1.4) | 6(1.5) | 9(1.3) | 10(1.4) | 5(0.6) | 5(0.6) | 16(0.4) | 8(0.1) | 3(0.0) | 2(0.0) | 69(0.3) |
| *C. glabrata* | 0(0.0) | 0(0.0) | 0(0.0) | 0(0.0) | 1(0.1) | 0(0.0) | 2(0.1) | 1(0.0) | 0(0.0) | 0(0.0) | 4(0.0) |
| ***Total*** | 369(100.0) | 389(100.0) | 684(100.0) | 698(100.0) | 819(100.0) | 811(100.0) | 3792(100.0) | 5757(100.0) | 8532(100.0) | 4618(100.0) | 26469(100.0) |
|  |  |  |  |  |  |  |  |  |  |  |  |

**3.2 Pathogens of lower respiratory tract infection and their gender and age distribution. Table2.**

Table2-1. Composition ratio of lower respiratory tract pathogens in children of different genders

|  | Infant(0~1years old) | | *P* | Toddler(1~3 years old ) | |  | Preschool age(3~6 years old ) | |  | School age(6~ years old ) | |
| --- | --- | --- | --- | --- | --- | --- | --- | --- | --- | --- | --- |
|  | male | female |  | male | female |  | male | female |  | male | female |
| ***G^-^*** | 6711(73.8) | 5434(78.7) |  | 2888(77.0) | 2118(75.9) |  | 1448(74.6) | 1185(73.5) |  | 174(80.2) | 132(80.0) |
| *H. influenzae* | 859(9.5) | 2740(39.7) | *<0.01* | 1503(40.1) | 1132(40.6) |  | 684(35.3) | 590(36.6) |  | 84(38.7) | 58(35.2) |
| *M. catarrhalis* | 455(5.0) | 1508(21.8) | *<0.01* | 1276(34.0) | 916(32.8) |  | 714(36.8) | 546(33.9) |  | 36(16.6) | 24(14.6) |
| *K. pneumoniae* | 2387(26.3) | 377(5.5) | *<0.01* | 26(0.7) | 14(0.5) |  | 17(0.9) | 12(0.7) |  | 19(8.8) | 15(9.1) |
| *E. coli* | 1383(15.2) | 338(4.9) | *<0.01* | 18(0.5) | 11(0.4) |  | 4(0.2) | 3(0.2) |  | 2(0.9) | 2(1.2) |
| *E. cloacae* | 453(5.0) | 101(1.5) | *<0.01* | 12(0.3) | 7(0.3) |  | 2(0.1) | 5(0.3) |  | 3(1.4) | 4(2.4) |
| *A. baumannii* | 331(3.6) | 49(0.7) | *<0.01* | 12(0.3) | 11(0.4) |  | 9(0.5) | 11(0.7) |  | 8(3.7) | 9(5.5) |
| *P. aeruginosa* | 160(1.8) | 93(1.4) | *<0.05* | 14(0.4) | 12(0.4) |  | 5(0.3) | 5(0.3) |  | 7(3.2) | 14(8.5) |
| *S. marcescens* | 162(1.8) | 49(0.7) | *<0.01* | 0(0.0) | 1(0.0) |  | 0(0.0) | 0(0.0) |  | 0(0.0) | 0(0.0) |
| *K. oxytoca* | 123(1.4) | 47(0.7) | *<0.01* | 4(0.1) | 3(0.1) |  | 2(0.1) | 0(0.0) |  | 2(0.9) | 1(0.6) |
| *K. aerogenes* | 122(1.3) | 37(0.5) | *<0.01* | 0(0.0) | 0(0.0) |  | 1(0.1) | 0(0.0) |  | 1(0.5) | 0(0.0) |
| *C. freundii* | 33(0.4) | 5(0.1) | *<0.01* | 0(0.0) | 0(0.0) |  | 1(0.1) | 1(0.1) |  | 0(0.0) | 0(0.0) |
| *R．plantarum* | 50(0.6) | 18(0.3) | *<0.01* | 0(0.0) | 0(0.0) |  | 1(0.1) | 0(0.0) |  | 0(0.0) | 0(0.0) |
| *S. maltophilia* | 77(0.9) | 19(0.3) | *<0.01* | 3(0.1) | 1(0.0) |  | 0(0.0) | 1(0.1) |  | 0(0.0) | 0(0.0) |
| *B. cepacia* | 7(0.1) | 14(0.2) |  | 6(0.2) | 1(0.0) |  | 2(0.1) | 1(0.1) |  | 3(1.4) | 1(0.6) |
| *Others* | 101(1.1) | 33(0.5) |  | 9(0.2) | 4(0.1) |  | 3(0.2) | 6(0.4) |  | 6(2.8) | 2(1.2) |
| ***G^+^*** | 2341(25.8) | 1459(21.1) |  | 859(22.9) | 669(24.0) |  | 492(25.4) | 426(26.4) |  | 43(19.8) | 33(20.0) |
| *S. pneumoniae* | 108(1.2) | 1027(14.9) | *<0.01* | 809(21.6) | 630(22.6) |  | 455(23.5) | 399(24.8) |  | 26(12.0) | 20(12.1) |
| *S. aureus* | 2072(22.8) | 421(6.1) | *<0.01* | 43(1.2) | 35(1.3) |  | 30(1.6) | 24(1.5) |  | 12(5.5) | 9(5.5) |
| *S.agalactiae* | 54(0.6) | 4(0.1) | *<0.01* | 0(0.0) | 0(0.0) |  | 1(0.1) | 0(0.0) |  | 0(0.0) | 0(0.0) |
| *S. pyogenes* | 0(0.0) | 2(0.0) |  | 3(0.1) | 0(0.0) |  | 5(0.3) | 2(0.1) |  | 3(1.4) | 3(1.8) |
| *S. haemolyticus* | 77(0.9) | 1(0.0) |  | 2(0.1) | 2(0.1) |  | 0(0.0) | 0(0.0) |  | 0(0.0) | 0(0.0) |
| *Others* | 26(0.3) | 3(0.0) |  | 2(0.1) | 0(0.0) |  | 0(0.0) | 0(0.0) |  | 0(0.0) | 0(0.0) |
| ***Fungi*** | 38(0.4) | 10(0.1) |  | 2(0.1) | 2(0.1) |  | 0(0.0) | 1(0.1) |  | 0(0.0) | 0(0.0) |
| *C. albicans* | 37(0.4) | 8(0.1) | *<0.01* | 2(0.1) | 2(0.1) |  | 0(0.0) | 1(0.1) |  | 0(0.0) | 0(0.0) |
| *C. glabrata* | 1(0.0) | 2(0.0) |  | 0(0.0) | 0(0.0) |  | 0(0.0) | 0(0.0) |  | 0(0.0) | 0(0.0) |
| ***Total*** | 9091(100.0) | 6905(100.0) |  | 3750(100.0) | 2789(100.0) |  | 1940(100.0) | 1612(100.0) |  | 217(100.0) | 165(100.0) |

Table2-2.Composition ratio of lower respiratory tract pathogens in children of different ages

|  | Infant(0~1years old) | Toddler(1~3years old) | Preschool age(3~6years old) | School age(6~years old) | *P* |
| --- | --- | --- | --- | --- | --- |
| ***G-*** | 12145 (75.9) | 5006 (76.6) | 2633 (74.1) | 306 (80.1) |  |
| *H.influenzae* | 3599 (22.5) | 2635 (40.3) | 1274 (35.9) | 142(37.2) | *<0.01* |
| *M.catarrhalis* | 1963 (12.3) | 2192 (33.5) | 1260 (35.5) | 60 (15.7) | *<0.01* |
| *K.pneumoniae* | 2764 (17.3) | 40 (0.6) | 29 (0.8) | 34 (8.9) | *<0.01* |
| *E.coli* | 1721 (10.8) | 29 (0.4) | 7 (0.2) | 4 (1.0) | *<0.01* |
| *E.cloacae* | 554 (3.5) | 19 (0.3) | 7 (0.2) | 7 (1.8) | *<0.01* |
| *A.baumannii* | 380(2.4) | 23(0.4) | 20 (0.6) | 17 (4.5) |  |
| *P.aeruginosa* | 253 (1.6) | 26 (0.4) | 10 (0.3) | 21 (5.5) |  |
| *S.marcescens* | 211 (1.3) | 1 (0.0) | 0 (0.0) | 0 (0.0) |  |
| *K.oxytoca* | 170 (1.1) | 7 (0.1) | 2 (0.1) | 3 (0.8) |  |
| *K.aerogenes* | 159 (1.0) | 0 (0.0) | 1 (0.0) | 1 (0.3) |  |
| *C.freundii* | 38 (0.2) | 0 (0.0) | 2 (0.1) | 0 (0.0) |  |
| *R.planticola* | 68 (0.4) | 0 (0.0) | 1 (0.0) | 0 (0.0) |  |
| *S.maltophilia* | 96 (0.6) | 4 (0.1) | 1 (0.0) | 0 (0.0) |  |
| *B.cepacia* | 21 (0.1) | 7 (0.1) | 3 (0.1) | 4 (1.0) |  |
| *other* | 134 (0.8) | 13(0.2) | 9 (0.3) | 8 (2.1) |  |
| ***G+*** | 3800 (23.8) | 1528 (23.4) | 918 (25.8) | 76 (19.9) |  |
| *S.pneumoniae* | 1135 (7.1) | 1439 (22.0) | 854 (24.0) | 46 (12.0) | *<0.01* |
| *S.aureus* | 2493 (15.6) | 78 (1.2) | 54 (1.5) | 21 (5.5) | *<0.01* |
| *S.agalactiae* | 58 (0.4) | 0 (0.4) | 1 (0.4) | 0 (0.4) |  |
| *S.pyogenes* | 2 (0.0) | 3 (0.0) | 7 (0.2) | 6 (1.6) |  |
| *S.haemolyticus* | 78 (0.5) | 4 (0.1) | 0 (0.0) | 0 (0.0) |  |
| *S.epidermidis* | 9 (0.1) | 0 (0.0) | 0 (0.0) | 0 (0.0) |  |
| *others* | 20 (0.1) | 2 (0.0) | 0 (0.0) | 0 (0.0) |  |
| ***Fungi*** | 48 (0.3) | 4 (0.1) | 1 (0.0) | 0 (0.0) |  |
| *C.albicans* | 45 (0.3) | 4 (0.1) | 1 (0.0) | 0 (0.0) |  |
| *C. glabrata* | 3 (0.0) | 0 (0.0) | 0 (0.0) | 0 (0.0) |  |
| ***Total*** | 15996 (100.0) | 6539 (100.0) | 3552 (100.0) | 382 (100.0) |  |

**3.3 Pathogens and seasonal distribution of lower respiratory tract infection. Table3.**

Table 3. Distribution and constituent ratio of pathogens in lower respiratory tract in different seasons isolated from 2018 to 2020 (%)

|  | 1-3months | 4-6 months | 7-9 months | 10-12 months | *P* |
| --- | --- | --- | --- | --- | --- |
| ***G^-^*** | 4173(80.8) | 3229(79.7) | 2893(75.8) | 4225(71.9) |  |
| *H. influenzae* | 2397(46.4) | 1479(36.5) | 756(19.8) | 915(15.6) | *<0.01* |
| *M. catarrhalis* | 1018(19.7) | 766(18.9) | 788(20.6) | 1814(30.9) | *<0.01* |
| *K. pneumoniae* | 224(4.3) | 359(8.9) | 559(14.6) | 258(4.4) |  |
| *E. coli* | 289(5.6) | 277(6.8) | 289(7.6) | 209(3.6) |  |
| *E. cloacae* | 64(1.2) | 85(2.1) | 129(3.4) | 57(1.0) |  |
| *A. baumannii* | 25(0.5) | 74(1.8) | 92(2.4) | 29(0.5) |  |
| *P. aeruginosa* | 31(0.6) | 37(0.9) | 67(1.8) | 53(0.9) |  |
| *S. marcescens* | 24(0.5) | 17(0.4) | 51(1.3) | 34(0.6) |  |
| *K. oxytoca* | 19(0.4) | 27(0.7) | 50(1.3) | 19(0.3) |  |
| *K. aerogenes* | 23(0.4) | 27(0.7) | 29(0.8) | 25(0.4) |  |
| *C. freundii* | 0(0.0) | 12(0.3) | 9(0.2) | 8(0.1) |  |
| *R. plantarum* | 14(0.3) | 16(0.4) | 14(0.4) | 4(0.1) |  |
| *S. maltophilia* | 8(0.2) | 17(0.4) | 12(0.3) | 5(0.1) |  |
| *B. cepacia* | 12(0.2) | 9(0.2) | 4(0.1) | 4(0.1) |  |
| *others* | 25(0.5) | 27(0.7) | 44(1.2) | 791(13.5) |  |
| ***G^+^*** | 984(19.1) | 820(20.2) | 920(24.1) | 1645(28.0) |  |
| *S. pneumoniae* | 605(11.7) | 416(10.3) | 456(11.9) | 1255(21.4) | *<0.01* |
| *S. aureus* | 357(6.9) | 381(9.4) | 444(11.6) | 375(6.4) |  |
| *S. agalactiae* | 6(0.1) | 10(0.2) | 9(0.2) | 5(0.1) |  |
| *S. pyogenes* | 3(0.1) | 4(0.1) | 3(0.1) | 3(0.1) |  |
| *S. haemolyticus* | 4(0.1) | 7(0.2) | 4(0.1) | 0(0.0) |  |
| *others* | 9(0.2) | 2(0.0) | 4(0.1) | 7(0.1) |  |
| ***Fungi*** | 5(0.1) | 3(0.1) | 2(0.1) | 4(0.1) |  |
| *C. albicans* | 5(0.1) | 3(0.1) | 1(0.0) | 4(0.1) |  |
| *C. glabrata* | 0(0.0) | 0(0.0) | 1(0.0) | 0(0.0) |  |
| ***total*** | 5164(100.0) | 4053(100.0) | 3817(100.0) | 5873(100.0) | */* |

**3.4 Pathogens of lower respiratory tract infection and pandemic of COVID-19 situation. Table4.**

Table 4 Distribution and constituent ratio of pathogens in lower respiratory tract in before and after the pandemic situation of COVID19 (%)

|  | 2018 | | | |  | 2019 | | | |  |  |  |  |  | 2020 | | |  |
| --- | --- | --- | --- | --- | --- | --- | --- | --- | --- | --- | --- | --- | --- | --- | --- | --- | --- | --- |
|  | 1-3  moths | 4-6  moths | 7-9  moths | 10-12 moths |  | 1-3  moths | 4-6  moths | 7-9  moths | 10-12 moths |  | 1-3  moths | *P* | 4-6  moths | *P* | 7-9  moths | *P* | 10-12 moths | *P* |
| ***G^-^*** | 1041  (79.8) | 1060  (76.4) | 870  (76.0) | 1394  (72.6) |  | 2062  (83.5) | 1700  (84.6) | 1221  (76.4) | 1792  (73.0) |  | 1070  (77.0) |  | 469  (71.6) |  | 802  (74.6)  89  (8.3)  344  (32.0)  139  (12.9)  80  (7.4)  41  (3.8)  18  (1.7)  14  (1.3)  17  (1.6)  9  (0.8)  0  (0.0)  3  (0.3)  3  (0.3)  1(0.1)  18  (1.7)  271  (25.2)  71  (6.6)  192  (17.9)  1  (0.1)  1  (0.1)  3  (0.3)  3  (0.3)  0  (0.0)  0  (0.0)  0  (0.0)  1075  (100.0) |  | 1039  (69.4) |  |
| *H. influenzae* | 643  (49.3) | 473  (34.1) | 265  (23.2) | 531  (27.7) |  | 1316  (53.3) | 922  (45.9) | 402  (25.2) | 192  (7.8) |  | 438  (31.5) | *<0.01* | 84  (12.8) | *<0.01* |  | *<0.01* | 192  (12.8) | *<0.01* |
| *M. catarrhalis* | 207  (15.9) | 259  (18.7) | 157  (13.7) | 526  (27.4) |  | 443  (17.9) | 369  (18.4) | 287  (18.0) | 644  (26.2) |  | 368  (26.5) | *<0.01* | 138  (21.1) | *>0.05* |  | *<0.01* | 644  (43.0) | *<0.01* |
| *K. pneumoniae* | 51  (3.9) | 134  (9.7) | 213  (18.6) | 112  (5.8) |  | 87  (3.5) | 139  (6.9) | 207  (13.0) | 73  (3.0) |  | 86  (6.2) |  | 86  (13.1) |  |  |  | 73  (4.9) |  |
| *E. coli* | 80  (6.1) | 80  (5.8) | 85  (7.4) | 103  (5.4) |  | 105  (4.3) | 123  (6.1) | 124  (7.8) | 53  (2.2) |  | 104  (7.5) |  | 74  (11.3) |  |  |  | 53  (3.5) |  |
| *E. cloacae* | 11  (0.8) | 31  (2.2) | 40  (3.5) | 37  (1.9) |  | 37  (1.5) | 33  (1.6) | 48  (3.0) | 10  (0.4) |  | 16  (1.2) |  | 21  (3.2) |  |  |  | 10  (0.7) |  |
| *P. aeruginosa* | 11  (0.8) | 13  (0.9) | 26  (2.3) | 21  (1.1) |  | 11  (0.4) | 18  (0.9) | 23  (1.4) | 16  (0.7) |  | 9  (0.6) |  | 6  (0.9) |  |  |  | 16  (1.1) |  |
| *S. marcescens* | 8  (0.6) | 3  (0.2) | 11  (1.0) | 14  (0.7) |  | 9  (0.4) | 8  (0.4) | 26  (1.6) | 10  (0.4) |  | 7  (0.5) |  | 6  (0.9) |  |  |  | 10  (0.7) |  |
| *K. oxytoca* | 2  (0.2) | 7  (0.5) | 11  (1.0) | 7  (0.4) |  | 9  (0.4) | 10  (0.5) | 22  (1.4) | 6  (0.2) |  | 8  (0.6) |  | 10  (1.5) |  |  |  | 6  (0.4) |  |
| *K. aerogenes* | 6  (0.5) | 7  (0.5) | 5  (0.4) | 13  (0.7) |  | 7  (0.3) | 15  (0.7) | 15  (0.9) | 6  (0.2) |  | 10  (0.7) |  | 5  (0.8) |  |  |  | 6  (0.4) |  |
| *C. freundii* | 0  (0.0) | 5  (0.4) | 5  (0.4) | 2  (0.1) |  | 0  (0.0) | 6  (0.3) | 4  (0.3) | 3  (0.1) |  | 0  (0.0) |  | 1  (0.2) |  |  |  | 3  (0.2) |  |
| *R. plantarum* | 1  (0.1) | 7  (0.5) | 4  (0.3) | 0  (0.0) |  | 7  (0.3) | 3  (0.1) | 7  (0.4) | 2  (0.1) |  | 6  (0.4) |  | 6  (0.9) |  |  |  | 2  (0.1) |  |
| *S. maltophilia* | 2  (0.2) | 6  (0.4) | 3  (0.3) | 3  (0.2) |  | 5  (0.2) | 10  (0.5) | 6  (0.4) | 1  (0.0) |  | 1  (0.1) |  | 1  (0.2) |  |  |  | 1  (0.1) |  |
| *B. cepacia* | 6(0.5) | 4(0.3) | 2(0.2) | 0(0.0) |  | 3(0.1) | 2(0.1) | 1(0.1) | 2(0.1) |  | 3(0.2) |  | 3(0.5) |  |  |  | 2(0.1) |  |
| *others* | 7  (0.5) | 6  (0.4) | 7  (0.6) | 12  (0.6) |  | 13  (0.5) | 12  (0.6) | 19  (1.2) | 766  (31.2) |  | 5  (0.4) |  | 9  (1.4) |  |  |  | 13  (0.9) |  |
| ***G^+^*** | 261  (20.0) | 326  (23.5) | 272  (23.8) | 524  (27.3) |  | 405  (16.4) | 310  (15.4) | 377  (23.6) | 663  (27.0) |  | 318  (22.9) |  | 184  (28.1) |  |  |  | 458  (30.6) |  |
| *S. pneumoniae* | 169  (13.0) | 204  (14.7) | 149  (13.0) | 416  (21.7) |  | 247  (10.0) | 158  (7.9) | 236  (14.8) | 520  (21.2) |  | 189  (13.6) | *<0.01* | 54  (8.2) | *<0.01* |  | *<0.01* | 319  (21.3) | *>0.05* |
| *S. aureus* | 87  (6.7) | 118  (8.5) | 117  (10.2) | 102  (5.3) |  | 148  (6.0) | 140  (7.0) | 135  (8.4) | 135  (5.5) |  | 122  (8.8) |  | 123  (18.8) |  |  |  | 138  (9.2) |  |
| *S. agalactiae* | 1  (0.1) | 3  (0.2) | 4  (0.3) | 3  (0.2) |  | 2  (0.1) | 1  (0.0) | 4  (0.3) | 2  (0.1) |  | 3  (0.2) |  | 6  (0.9) |  |  |  | 0  (0.0) |  |
| *S. pyogenes* | 0  (0.0) | 1  (0.1) | 1  (0.1) | 1  (0.1) |  | 2  (0.1) | 3  (0.1) | 1  (0.1) | 2  (0.1) |  | 1  (0.1) |  | 0  (0.0) |  |  |  | 0  (0.0) |  |
| *S. haemolyticus* | 3  (0.2) | 0  (0.0) | 0  (0.0) | 0  (0.0) |  | 0  (0.0) | 6  (0.3) | 1  (0.1) | 0  (0.0) |  | 1  (0.1) |  | 1  (0.2) |  |  |  | 0  (0.0) |  |
| *others* | 1  (0.1) | 0  (0.0) | 1  (0.1) | 2  (0.1) |  | 6  (0.2) | 2  (0.1) | 0  (0.0) | 4  (0.2) |  | 2  (0.1) |  | 0  (0.0) |  |  |  | 1  (0.1) |  |
| ***Fungi*** | 3  (0.2) | 2  (0.1) | 2  (0.2) | 2  (0.1) |  | 2  (0.1) | 0  (0.0) | 0  (0.0) | 1  (0.0) |  | 0  (0.0) |  | 1  (0.2) |  |  |  | 1  (0.1) |  |
| *C. albicans* | 3  (0.2) | 2  (0.1) | 1  (0.1) | 2  (0.1) |  | 2  (0.1) | 0  (0.0) | 0  (0.0) | 1  (0.0) |  | 0  (0.0) |  | 1  (0.2) |  |  |  | 1  (0.1) |  |
| *C. glabrata* | 0  (0.0) | 0  (0.0) | 1  (0.1) | 0  (0.0) |  | 0  (0.0) | 0  (0.0) | 0  (0.0) | 0  (0.0) |  | 0  (0.0) |  | 0  (0.0) |  |  |  | 0  (0.0) |  |
| ***Total*** | 1305  (100.0) | 1388  (100.0) | 1144  (100.0) | 1920  (100.0) |  | 2469  (100.0) | 2010  (100.0) | 1598  (100.0) | 2455  (100.0) |  | 1390  (100.0) |  | 655  (100.0) |  |  |  | 1498  (100.0) |  |

**3.5 Pathogens and department distribution of lower respiratory tract infection. Table5.**

Table 5 Distribution and constituent ratio of pathogens in lower respiratory tract in different departments isolated from 2018 to 2020 (%)

|  | Pediatric Gastroenterology | Pediatric Cardiology | Pediatric Hematology and Oncology | NICU | PICU | Pediatric Rheumatology and Immunology | Pediatric Respiratory Medicine | Pediatric Neurological Rehabilitation | Pediatric nephrology | *P* |
| --- | --- | --- | --- | --- | --- | --- | --- | --- | --- | --- |
| ***G^-^*** | 974(75.6) | 1397(74.9) | 882(74.1) | 918(85.1) | 826(79.9) | 990(74.9) | 3846(75.9) | 1057(74.9) | 751(74.4) | *<0.01* |
| *H. influenzae* | 460(35.7) | 659(35.3) | 443(37.2) | 55(5.1) | 157(15.2) | 471(35.7) | 1639(32.4) | 458(32.5) | 351(34.8) | *<0.01* |
| *M. catarrhalis* | 306(23.8) | 568(30.5) | 357(30.0) | 28(2.6) | 75(7.3) | 386(29.2) | 1471(29.0) | 463(32.8) | 286(28.3) | *<0.01* |
| *K. pneumoniae* | 80(6.2) | 55(2.9) | 35(2.9) | 371(34.4) | 228(22.1) | 42(3.2) | 245(4.8) | 49(3.5) | 28(2.8) | *<0.01* |
| *E. coli* | 47(3.6) | 47(2.5) | 14(1.2) | 223(20.7) | 130(12.6) | 36(2.7) | 202(4.0) | 36(2.6) | 32(3.2) | *<0.01* |
| *E. cloacae* | 20(1.6) | 22(1.2) | 9(0.8) | 72(6.7) | 58(5.6) | 15(1.1) | 70(1.4) | 7(0.5) | 16(1.6) |  |
| *A. baumannii* | 7(0.5) | 7(0.4) | 5(0.4) | 60(5.6) | 24(2.3) | 8(0.6) | 36(0.7) | 4(0.3) | 7(0.7) | *<0.01* |
| *P. aeruginosa* | 13(1.0) | 12(0.6) | 10(0.8) | 20(1.9) | 34(3.3) | 8(0.6) | 47(0.9) | 11(0.8) | 4(0.4) |  |
| *S. marcescens* | 8(0.6) | 6(0.3) | 1(0.1) | 20(1.9) | 23(2.2) | 7(0.5) | 32(0.6) | 8(0.6) | 8(0.8) |  |
| *K. oxytoca* | 6(0.5) | 3(0.2) | 1(0.1) | 13(1.2) | 27(2.6) | 6(0.5) | 29(0.6) | 9(0.6) | 2(0.2) |  |
| *K. aerogenes* | 9(0.7) | 5(0.3) | 2(0.2) | 16(1.5) | 16(1.5) | 4(0.3) | 20(0.4) | 3(0.2) | 7(0.7) |  |
| *C. freundii* | 2(0.2) | 2(0.1) | 0(0.0) | 3(0.3) | 4(0.4) | 0(0.0) | 4(0.1) | 1(0.1) | 2(0.2) |  |
| *R. plantarum* | 4(0.3) | 4(0.2) | 1(0.1) | 8(0.7) | 6(0.6) | 0(0.0) | 12(0.2) | 2(0.1) | 1(0.1) |  |
| *S. maltophilia* | 6(0.5) | 0(0.0) | 0(0.0) | 10(0.9) | 8(0.8) | 0(0.0) | 5(0.1) | 1(0.1) | 1(0.1) |  |
| *B. cepacia* | 0(0.0) | 0(0.0) | 1(0.1) | 2(0.2) | 16(1.5) | 0(0.0) | 0(0.0) | 0(0.0) | 0(0.0) |  |
| *others* | 6(0.5) | 7(0.4) | 3(0.3) | 17(1.6) | 20(1.9) | 7(0.5) | 34(0.7) | 5(0.4) | 6(0.6) |  |
| ***G^+^*** | 314(24.4) | 467(25.0) | 308(25.9) | 160(14.8) | 205(19.8) | 331(25.1) | 1218(24.0) | 354(25.1) | 258(25.6) | *<0.01* |
| *S. pneumoniae* | 210(16.3) | 323(17.3) | 268(22.5) | 7(0.6) | 57(5.5) | 222(16.8) | 826(16.3) | 254(18.0) | 173(17.1) | *<0.01* |
| *S. aureus* | 103(8.0) | 141(7.6) | 37(3.1) | 118(10.9) | 148(14.3) | 106(8.0) | 384(7.6) | 97(6.9) | 82(8.1) | *<0.01* |
| *S. agalactiae* | 0(0.0) | 0(0.0) | 0(0.0) | 16(1.5) | 0(0.0) | 0(0.0) | 2(0.0) | 1(0.1) | 1(0.1) |  |
| *S. pyogenes* | 0(0.0) | 2(0.1) | 3(0.3) | 0(0.0) | 0(0.0) | 2(0.2) | 2(0.0) | 1(0.1) | 1(0.1) |  |
| *S. haemolyticus* | 0(0.0) | 0(0.0) | 0(0.0) | 9(0.8) | 0(0.0) | 0(0.0) | 1(0.0) | 0(0.0) | 0(0.0) |  |
| *others* | 1(0.1) | 1(0.1) | 0(0.0) | 10(1.0) | 0(0.0) | 1(0.1) | 3(0.1) | 1(0.1) | 1(0.1) |  |
| ***Fungi*** | 0(0.0) | 1(0.1) | 0(0.0) | 1(0.1) | 3(0.3) | 0(0.0) | 1(0.0) | 0(0.0) | 0(0.0) |  |
| *C. albicans* | 0(0.0) | 1(0.1) | 0(0.0) | 1(0.1) | 2(0.2) | 0(0.0) | 1(0.0) | 0(0.0) | 0(0.0) |  |
| *C. glabrata* | 0(0.0) | 0(0.0) | 0(0.0) | 0(0.0) | 1(0.1) | 0(0.0) | 0(0.0) | 0(0.0) | 0(0.0) |  |
| ***Total*** | 1288(100.0) | 1865(100.0) | 1190(100.0) | 1079(100.0) | 1034(100.0) | 1321(100.0) | 5065(100.0) | 1411(100.0) | 1009(100.0) | */* |

**3.6 Analysis of drug resistance of *S. pneumoniae.* Table6, Table7.**

Table 6 Distribution of drug resistance of *S. pneumoniae* from 2018 to 2020

| *S. pneumoniae*（2732strains） | | |
| --- | --- | --- |
| Antibiotic | Infection site | Resistance rate(%) |
| PEN | others | 0.2 |
| PEN | Meningitis | 88.7 |
| PEN | oral | 33.6 |
| AMX | others | 31.7 |
| CRO | others | 15.8 |
| CRO | Meningitis | 20.4 |
| CTX | Meningitis | 24.6 |
| CTX | others | 14.2 |
| MEM |  | 18.7 |
| RIF |  | 1.8 |
| CLX |  | 0 |
| LVX |  | 0.1 |
| MFX |  | 0 |
| OFX |  | 0.3 |
| SXT |  | 84.2 |
| CLI |  | 99.4 |
| LIN |  | 0 |
| ERY |  | 99 |
| TLT |  | 0.1 |
| LNZ |  | 0 |
| VAN |  | 0 |
| CHL |  | 11.3 |
| QDA |  | 9.7 |
| TCY |  | 91.6 |

Table 7 Susceptibility results of *S. pneumoniae* from 2011 to 2020

| Antibiotics | Infection site | Changes in drug resistance of *S. pneumoniae* from 2011 to 2020(%) | | | | | | | | | |
| --- | --- | --- | --- | --- | --- | --- | --- | --- | --- | --- | --- |
|  |  | 2011 | 2012 | 2013 | 2014 | 2015 | 2016 | 2017 | 2018 | 2019 | 2020 |
| PEN | others | 0.0 | 0.0 | 0.0 | 0.0 | 0.0 | 0.0 | 0.4 | 0.5 | 0.0 | 0.0 |
| PEN | Meningitis | 80.5 | 80.5 | 80.7 | 80.8 | 82.4 | 89.5 | 91.1 | 90.6 | 88.5 | 86.5 |
| PEN | oral | 41.9 | 41.3 | 42.3 | 42.3 | 41.2 | 73.7 | 55.1 | 27.2 | 35.5 | 40.2 |
| AMX | others | 5.4 | 5.2 | 5.7 | 5.9 | 25.0 | 47.4 | 31.8 | 32.3 | 35.4 | 24.3 |
| CTX | others | 3.9 | 4.1 | 3.8 | 3.8 | 11.8 | 21.1 | 14.3 | 12.9 | 16.4 | 22.6 |
| CTX | Meningitis | 15.0 | 14.9 | 15.2 | 15.4 | 11.8 | 31.6 | 23.1 | 22.5 | 27.2 | 12.3 |
| MEM | others | 11.3 | 11.6 | 11.5 | 11.5 | 17.2 | 26.3 | 17.1 | 15.7 | 23.2 | 14.9 |
| LVX |  | 3.9 | 3.7 | 3.8 | 3.8 | 0.0 | 0.0 | 0.0 | 0.2 | 0.0 | 0.0 |
| MFX |  | 0.0 | 0.0 | 0.0 | 0.0 | 0.0 | 0.0 | 0.0 | 0.0 | 0.0 | 0.0 |
| OFX |  | 4.0 | 3.7 | 3.9 | 3.8 | 0.0 | 0.0 | 0.0 | 0.2 | 0.4 | 0.3 |
| SXT |  | 81.2 | 81.4 | 81.0 | 80.8 | 82.4 | 94.7 | 82.9 | 85.9 | 83.7 | 82.2 |
| ERY |  | 100.0 | 100.0 | 100.0 | 100.0 | 100.0 | 100.0 | 99.4 | 99.1 | 99.0 | 99.0 |
| LNZ |  | 0.0 | 0.0 | 0.0 | 0.0 | 0.0 | 0.0 | 0.0 | 0.0 | 0.0 | 0.0 |
| VAN |  | 0.0 | 0.0 | 0.0 | 0.0 | 0.0 | 0.0 | 0.0 | 0.0 | 0.0 | 0.0 |
| CHL |  | 3.9 | 3.6 | 3.8 | 3.8 | 29.4 | 15.8 | 9.0 | 10.2 | 12.6 | 10.7 |
| TCY |  | 88.7 | 88.4 | 88.7 | 88.5 | 76.5 | 100.0 | 94.6 | 93.5 | 91.1 | 89.6 |

**3.7 Analysis of drug resistance of *S. aureus*. Table8, Table9.**

Table 8 Distribution of drug resistance of *S. aureus* from 2018 to 2020

| Antibiotics | *S. aureus*(1557 strains) | |
| --- | --- | --- |
|  | MRSA(416strains27%) | MSSA(1141strains73%) |
| PEN | 100.0 | 87.7 |
| OXA | 100.0 | 0.0 |
| GEN | 1.9 | 10.8 |
| RIF | 1.2 | 0.1 |
| CIP | 3.1 | 5.7 |
| LVX | 3.1 | 5.6 |
| MFX | 2.2 | 4.7 |
| SXT | 4.1 | 16.5 |
| CLIN | 78.7 | 38.6 |
| ERY | 80.6 | 45.8 |
| NIT | 0.2 | 0.0 |
| LNZ | 0.0 | 0.0 |
| VAN | 0.2 | 0.0 |
| QDA | 8.4 | 21.1 |
| TCY | 35.4 | 12.1 |
| TGC | 0.0 | 0.0 |

Table 9 Susceptibility results of *S. aureus* from 2011 to 2020

| Antibiotics | Changes in drug resistance of *s. aureus* from 2011 to 2020(%) | | | | | | | | | |
| --- | --- | --- | --- | --- | --- | --- | --- | --- | --- | --- |
|  | 2011 | 2012 | 2013 | 2014 | 2015 | 2016 | 2017 | 2018 | 2019 | 2020 |
| PEN | 92.3 | 92.2 | 93.4 | 93.3 | 91.5 | 94.0 | 90.7 | 92.9 | 90.6 | 89.8 |
| OXA | 17.9 | 18.1 | 18.2 | 19.2 | 22.1 | 23.2 | 20.5 | 30.0 | 25.9 | 24.6 |
| GEN | 10.2 | 12.1 | 12.1 | 13.5 | 10.4 | 13.2 | 12.2 | 8.8 | 9.2 | 7.2 |
| RIF | 0.3 | 0.2 | 0.4 | 1.0 | 0.0 | 0.0 | 0.2 | 0.2 | 0.4 | 0.5 |
| CIP | 4.9 | 5.2 | 4.9 | 4.8 | 7.8 | 7.3 | 3.7 | 4.7 | 4.9 | 5.4 |
| LVX | 4.7 | 4.6 | 4.2 | 4.8 | 7.1 | 6.0 | 3.9 | 4.5 | 4.9 | 5.3 |
| MFX | 2.9 | 3.2 | 3.3 | 2.9 | 3.9 | 4.6 | 3.1 | 4.3 | 3.4 | 4.4 |
| SXT | 19.2 | 19.3 | 18.9 | 19.4 | 17.7 | 21.4 | 16.9 | 13.0 | 13.4 | 13.1 |
| CLI | 54.9 | 54.8 | 55.2 | 55.8 | 44.8 | 36.4 | 32.2 | 44.1 | 51.3 | 51.5 |
| ERY | 56.2 | 56.3 | 56.5 | 56.7 | 56.5 | 56.3 | 56.2 | 57.6 | 54.0 | 54.3 |
| NIT | 0.0 | 0.0 | 0.0 | 0.0 | 0.0 | 0.0 | 0.0 | 0.0 | 0.2 | 0.0 |
| LNZ | 0.0 | 0.0 | 0.0 | 0.0 | 0.0 | 0.0 | 0.0 | 0.0 | 0.0 | 0.0 |
| VAN | 0.0 | 0.0 | 0.0 | 0.0 | 0.0 | 0.0 | 0.0 | 0.0 | 0.4 | 0.0 |
| QDA | 19.7 | 19.8 | 20.8 | 21.2 | 6.5 | 0.0 | 0.0 | 7.8 | 20.9 | 22.1 |
| TCY | 20.2 | 19.9 | 20.3 | 20.2 | 26.6 | 19.9 | 20.0 | 22.3 | 19.1 | 15.1 |
| TGC | 0.0 | 0.0 | 0.0 | 0.0 | 0.0 | 0.0 | 0.0 | 0.0 | 0.0 | 0.0 |

**3.8 Analysis of drug resistance of *H. influenzae* and *M. catarrhalis* Table10, Table11, Table12.**

Table 10 Distribution of drug resistance of *H. influenzae* and *M. catarrhalis* from 2018 to 2020

| Antibiotics | *H. influenzae*(5547strains) | *M. catarrhalis* (4386 strains) |
| --- | --- | --- |
| AMP | 91.1 | 98.9 |
| AMC | 28 | 0.7 |
| CXM | 65 | 6.4 |
| CTX | 0.0 | 0.0 |
| CEC | 72.2 | 11.7 |
| RIF | 0.1 | 0.0 |
| OFX | 0.0 | 0.0 |
| SXT | 67.7 | 17.9 |
| CHL | 4.1 | 0.5 |
| TCY | 4.3 | 1.3 |

Table 11 Susceptibility results of *H. influenzae* from 2011 to 2020

| Antibiotics | Changes in drug resistance of *H. influenzae* from 2011 to 2020(%) | | | | | | | | | |
| --- | --- | --- | --- | --- | --- | --- | --- | --- | --- | --- |
|  | 2011 | 2012 | 2013 | 2014 | 2015 | 2016 | 2017 | 2018 | 2019 | 2020 |
| AMP | 64.8 | 64.9 | 65.3 | 65.6 | 82.2 | 91.1 | 92.4 | 91.9 | 91.7 | 86.5 |
| AMC | 8.6 | 7.5 | 9.2 | 6.5 | 13.3 | 20.0 | 27.0 | 38.6 | 27.3 | 9.3 |
| CXM | 37.2 | 37.9 | 38.4 | 40.6 | 55.6 | 71.1 | 78.8 | 71.3 | 64.3 | 53.2 |
| CTX | 0.0 | 0.0 | 0.0 | 0.0 | 0.0 | 0.0 | 0.0 | 0.0 | 0.0 | 0.0 |
| CEC | 34.2 | 34.9 | 36.8 | 38.7 | 60.0 | 77.8 | 85.6 | 79.8 | 70.6 | 57.5 |
| RIF | 0.0 | 0.0 | 0.0 | 0.0 | 0.0 | 0.0 | 0.0 | 0.2 | 0.1 | 0.0 |
| OFX | 0.0 | 0.0 | 0.0 | 0.0 | 0.0 | 0.0 | 0.0 | 0.0 | 0.0 | 0.0 |
| SXT | 68.2 | 68.9 | 70.2 | 71.9 | 82.2 | 75.6 | 72.2 | 70.5 | 67.5 | 56.9 |
| CHL | 9.3 | 9.2 | 9.2 | 9.4 | 11.1 | 1.2 | 4.1 | 3.7 | 3.8 | 4.5 |
| TCY | 12.4 | 12.3 | 12.2 | 12.5 | 8.9 | 2.2 | 4.8 | 4.6 | 4.0 | 3.6 |

Table 12 Susceptibility results of *M. catarrhalis* from 2011 to 2020 M.

| Antibiotics | Changes in drug resistance of *M. catarrhalis* from 2011 to 2020(%) | | | | | | | | | |
| --- | --- | --- | --- | --- | --- | --- | --- | --- | --- | --- |
|  | 2011 | 2012 | 2013 | 2014 | 2015 | 2016 | 2017 | 2018 | 2019 | 2020 |
| AMP | 97.4 | 98.2 | 96.2 | 96.4 | 98.6 | 100.0 | 98.4 | 99.0 | 98.6 | 99.2 |
| AMC | 6.2 | 7.1 | 6.5 | 7.7 | 6.2 | 1.2 | 0.4 | 0.8 | 0.9 | 0.3 |
| CXM | 20.2 | 21.2 | 22.7 | 23.1 | 43.8 | 44.2 | 27.0 | 11.5 | 6.5 | 3.5 |
| CTX | 0.0 | 0.0 | 0.0 | 0.0 | 0.0 | 0.0 | 0.0 | 0.0 | 0.0 | 0.0 |
| CEC | 20.1 | 22.1 | 23.1 | 23.1 | 37.5 | 41.2 | 40.9 | 21.4 | 11.7 | 7.0 |
| RIF | 6.2 | 6.5 | 6.9 | 7.7 | 0.0 | 0.0 | 0.0 | 0.1 | 0.1 | 0.0 |
| OFX | 0.0 | 0.0 | 0.0 | 0.0 | 0.0 | 0.0 | 0.0 | 0.0 | 0.0 | 0.0 |
| SXT | 56.2 | 58.4 | 59.2 | 61.5 | 81.2 | 94.7 | 29.7 | 23.4 | 18.6 | 12.0 |
| CHL | 5.2 | 6.2 | 5.9 | 7.7 | 0.0 | 0.0 | 0.0 | 0.3 | 0.4 | 0.7 |
| TCY | 0.0 | 0.0 | 0.0 | 0.0 | 0.0 | 10.5 | 1.5 | 1.4 | 1.7 | 0.7 |

**3.9 Analysis of drug resistance of *E. coli* and *K. pneumoniae*. Table13, Table14, Table15.**

Table 13 Distribution of drug resistance of *E. coli* and *K. pneumoniae* from 2018 to 2020

|  | *E. coli*(1064strains) | | |  | *K. pneumoniae*(1400strains) | | |
| --- | --- | --- | --- | --- | --- | --- | --- |
| Antibiotics | ESBL Negative  (618strains58%) | ESBL positive  (423 strains 40%) | CRE  (23 strains 2%) |  | ESBL Negative  (603 strains 43%) | ESBL positive  (385 strains 28%) | CRE  (402 strains 29%) |
| AMP | 61.3 | 100.0 | 100.0 |  | 66.3 | 100.0 | 100.0 |
| PIP | 59.1 | 98.8 | 100.0 |  | 6.0 | 100.0 | 100.0 |
| AMC | 3.3 | 6.2 | 100.0 |  | 4.7 | 25.7 | 100.0 |
| SAM | 27.8 | 52.6 | 100.0 |  | 9.9 | 90.5 | 100.0 |
| TZP | 0.3 | 1.4 | 100.0 |  | 0.2 | 38.1 | 100.0 |
| CZO | 40.5 | 100.0 | 100.0 |  | 10.6 | 100.0 | 100.0 |
| CXM | 1.5 | 99.4 | 100.0 |  | 6.1 | 98.1 | 100.0 |
| CAZ | 0.2 | 22.1 | 100.0 |  | 1.0 | 66.9 | 100.0 |
| CRO | 0.0 | 100.0 | 100.0 |  | 0.0 | 99.7 | 100.0 |
| CTX | 0.0 | 100.0 | 100.0 |  | 0.5 | 100.0 | 100.0 |
| FEP | 0.0 | 10.9 | 91.3 |  | 0.0 | 47.8 | 100.0 |
| CTT | 0.0 | 0.2 | 100.0 |  | 0.0 | 0.3 | 100.0 |
| CPD | 1.5 | 100.0 | 100.0 |  | 3.6 | 100.0 | 100.0 |
| CXA | 1.1 | 99.1 | 100.0 |  | 4.7 | 97.3 | 100.0 |
| ATM | 0.0 | 45.8 | 53.3 |  | 0.2 | 72.5 | 89.8 |
| DOR | 0.0 | 0.0 | 100.0 |  | 0.0 | 0.5 | 99.7 |
| IPM | 0.0 | 0.0 | 100.0 |  | 0.0 | 0.0 | 100.0 |
| MEM | 0.0 | 0.0 | 100.0 |  | 0.0 | 0.3 | 100.0 |
| AMK | 0.0 | 0.5 | 4.0 |  | 0.0 | 0.7 | 1.0 |
| GEN | 21.3 | 24.9 | 34.1 |  | 1.1 | 23.5 | 1.8 |
| TOB | 0.5 | 7.2 | 25.8 |  | 0.2 | 6.3 | 1.2 |
| NAL | 63.6 | 76.1 | 61.1 |  | 0.3 | 9.9 | 2.2 |
| CIP | 20.2 | 46.8 | 58.9 |  | 5.1 | 30.2 | 5.0 |
| LVX | 12.2 | 40.5 | 58.9 |  | 0.5 | 8.8 | 3.6 |
| MFX | 25.4 | 49.6 | 83.3 |  | 11.7 | 52.6 | 7.3 |
| SXT | 37.7 | 51.1 | 71.2 |  | 8.3 | 42.6 | 6.6 |
| NIT | 0.2 | 0.5 | 34.8 |  | 12.8 | 35.7 | 65.5 |
| TCY | 57.0 | 67.0 | 100.0 |  | 16.0 | 69.2 | 39.6 |

Table 14 Susceptibility results of *E. coli* from 2011 to 2020

| Antibiotics | Changes in drug resistance of *E. coil* from 2011 to 2020(%) | | | | | | | | | |
| --- | --- | --- | --- | --- | --- | --- | --- | --- | --- | --- |
|  | 2011 | 2012 | 2013 | 2014 | 2015 | 2016 | 2017 | 2018 | 2019 | 2020 |
| AMP | 80.2 | 81.2 | 80.9 | 81.4 | 83.7 | 78.7 | 84.9 | 77.9 | 79.8 | 73.2 |
| SAM | 39.1 | 39.5 | 38.7 | 38.9 | 44.4 | 31.5 | 49.4 | 37.4 | 42.8 | 38.9 |
| TZP | 0.8 | 0.8 | 0.9 | 0.9 | 1.6 | 0.9 | 0.4 | 1.8 | 3.5 | 2.3 |
| CZO | 45.5 | 45.6 | 47.8 | 42.5 | 68.9 | 100.0 | 100.0 | 55.6 | 47.0 | 47.4 |
| CAZ | 7.8 | 8.5 | 7.8 | 7.1 | 10.5 | 10.1 | 12.6 | 11.8 | 12.0 | 8.5 |
| CRO | 42.1 | 43.2 | 40.1 | 38.9 | 50.0 | 47.2 | 49.8 | 43.1 | 43.2 | 34.6 |
| FEP | 4.8 | 4.7 | 5.2 | 5.3 | 4.0 | 2.2 | 6.3 | 7.4 | 6.7 | 4.2 |
| CTT | 0.2 | 0.3 | 0.1 | 0.0 | 0.8 | 0.0 | 0.4 | 2.1 | 2.8 | 1.6 |
| ATM | 14.8 | 15.4 | 15.2 | 15.9 | 24.2 | 28.1 | 22.6 | 20.1 | 19.1 | 17.6 |
| ETP | 0 | 0 | 0 | 0 | 0 | 0 | 0 | 0 | 0 | 0 |
| IPM | 1.1 | 0.9 | 0.8 | 0.8 | 0.8 | 0.9 | 0.8 | 2.1 | 2.5 | 1.6 |
| AMK | 1.3 | 1.4 | 1.5 | 0.0 | 1.6 | 1.1 | 0.8 | 0.3 | 0.2 | 0.7 |
| GEN | 27.5 | 26.4 | 27.1 | 35.4 | 26.6 | 28.1 | 27.2 | 23.8 | 25.9 | 20.0 |
| TOB | 3.2 | 3.4 | 3.6 | 3.6 | 3.2 | 3.4 | 5.9 | 3.5 | 3.2 | 4.2 |
| CIP | 35.2 | 34.2 | 36.7 | 37.7 | 36.9 | 30.3 | 34.3 | 37.1 | 27.2 | 31.0 |
| LVX | 27.4 | 28.1 | 28.4 | 27.4 | 30.0 | 24.1 | 25.2 | 28.6 | 21.2 | 23.3 |
| SXT | 46.5 | 47.4 | 46.9 | 45.1 | 49.0 | 44.9 | 47.2 | 41.0 | 46.7 | 44.0 |
| NIT | 0.0 | 0.0 | 0.0 | 1.0 | 0.0 | 0.0 | 0.0 | 0.0 | 0.2 | 0.7 |

Table 15 Susceptibility results of *K. pneumoniae* from 2011 to 2020

| Antibiotics | Changes in drug resistance of *K. pneumoniae* l from 2011 to 2020(%) | | | | | | | | | |
| --- | --- | --- | --- | --- | --- | --- | --- | --- | --- | --- |
|  | 2011 | 2012 | 2013 | 2014 | 2015 | 2016 | 2017 | 2018 | 2019 | 2020 |
| AMP | 91.2 | 90.2 | 90.8 | 90.8 | 92.2 | 92.8 | 90.1 | 87.4 | 85.4 | 83.2 |
| SAM | 76.2 | 74.5 | 77.2 | 75.9 | 78.1 | 83.7 | 71.5 | 59.7 | 60.5 | 53.2 |
| TZP | 14.2 | 15.2 | 16.3 | 16.7 | 17.4 | 32 | 38.5 | 21.6 | 35.6 | 36.8 |
| CZO | 78.2 | 78.1 | 79.5 | 80.3 | 91.5 | 92.1 | 85.2 | 82.3 | 83.2 | 81.1 |
| CAZ | 76.2 | 74.2 | 75.9 | 74.1 | 75.2 | 78.1 | 57.1 | 48.3 | 49.4 | 45.1 |
| CRO | 76.4 | 78.1 | 79.2 | 79.4 | 79.6 | 84.6 | 70.4 | 57 | 60.1 | 50.9 |
| FEP | 58.7 | 57.7 | 58.1 | 59.6 | 68.5 | 72.7 | 51 | 39.5 | 43.8 | 41.1 |
| CTT | 8.2 | 8.4 | 8.1 | 8.8 | 0.4 | 1.3 | 16.7 | 19.6 | 34.6 | 34.8 |
| ATM | 78.7 | 76.4 | 76.6 | 75.4 | 76.3 | 79.3 | 51.6 | 44.8 | 48.2 | 45.1 |
| ETP | 0 | 0 | 0 | 0 | 0 | 0 | 0.8 | 0.3 | 0.3 | 0.4 |
| IPM | 0 | 0 | 0 | 0 | 0 | 0.9 | 16.7 | 19.6 | 35.7 | 34.7 |
| AMK | 0 | 0 | 0 | 0 | 0 | 0 | 0.4 | 1 | 0.4 | 0 |
| GEN | 12.4 | 14.2 | 13.9 | 13.6 | 5.2 | 13.5 | 28.5 | 7.3 | 9.4 | 5.3 |
| TOB | 0.4 | 0.6 | 0.9 | 1.3 | 0 | 0 | 3 | 3.7 | 1.8 | 0.5 |
| CIP | 6.1 | 5.6 | 7.2 | 6.7 | 5.7 | 5.6 | 14.6 | 12.4 | 12 | 10.4 |
| LVX | 1.2 | 1.4 | 1.1 | 1.8 | 0 | 0.3 | 3.2 | 3.1 | 3.7 | 3.8 |
| SXT | 61.1 | 60.5 | 60.1 | 59.9 | 62 | 15.9 | 18.3 | 17.8 | 20.7 | 10.2 |
| NIT | 11.9 | 11.8 | 12.7 | 13.7 | 80 | 45.8 | 23 | 46.2 | 30.7 | 31.9 |
